# Decoding YAP dependent transcription in the liver

**DOI:** 10.1093/nar/gkac624

**Published:** 2022-07-25

**Authors:** Francesca Biagioni, Ottavio Croci, Silvia Sberna, Elisa Donato, Arianna Sabò, Andrea Bisso, Laura Curti, Arianna Chiesa, Stefano Campaner

**Affiliations:** Center for Genomic Science of CGS@SEMM, Fondazione Istituto Italiano di Tecnologia (IIT), Milan, Italy; Center for Genomic Science of CGS@SEMM, Fondazione Istituto Italiano di Tecnologia (IIT), Milan, Italy; Center for Genomic Science of CGS@SEMM, Fondazione Istituto Italiano di Tecnologia (IIT), Milan, Italy; Center for Genomic Science of CGS@SEMM, Fondazione Istituto Italiano di Tecnologia (IIT), Milan, Italy; Department of Experimental Oncology, European Institute of Oncology (IEO)‐IRCCS, Milan, Italy; Department of Experimental Oncology, European Institute of Oncology (IEO)‐IRCCS, Milan, Italy; Center for Genomic Science of CGS@SEMM, Fondazione Istituto Italiano di Tecnologia (IIT), Milan, Italy; Center for Genomic Science of CGS@SEMM, Fondazione Istituto Italiano di Tecnologia (IIT), Milan, Italy; Center for Genomic Science of CGS@SEMM, Fondazione Istituto Italiano di Tecnologia (IIT), Milan, Italy

## Abstract

The transcriptional coactivator YAP is emerging as a master regulator of cell growth. In the liver, YAP activity is linked to hepatomegaly, regeneration, dedifferentiation, and aggressive tumor growth. Here we present genomic studies to address how YAP may elicit such profound biological changes in murine models. YAP bound the genome in a TEAD-dependent manner, either at loci constitutively occupied by TEAD or by pioneering enhancers, which comprised a fraction of HNF4a/FOXA-bound embryonic enhancers active during embryonic development but silent in the adult. YAP triggered transcription on promoters by recruiting BRD4, enhancing H3K122 acetylation, and promoting RNApol2 loading and pause-release. YAP also repressed HNF4a target genes by binding to their promoters and enhancers, thus preventing RNApol2 pause-release. YAP activation led to the induction of hepatocyte proliferation, accompanied by tissue remodeling, characterized by polarized macrophages, exhausted T-lymphocytes and dedifferentiation of endothelial cells into proliferative progenitors. Overall, these analyses suggest that YAP is a master regulator of liver function that reshapes the enhancer landscape to control transcription of genes involved in metabolism, proliferation, and inflammation, subverts lineage specification programs by antagonizing HNF4a and modulating the immune infiltrate and the vascular architecture of the liver.

## INTRODUCTION

The Yes-associated protein (YAP) is a transcriptional coactivator which, along with its paralogue TAZ (WWTR1), is the vertebrate effector of the Hippo pathway (1–3). In addition, YAP and TAZ can be regulated by WNT-signalling and cytoskeletal tension (4,5). Activation of YAP/TAZ, either by Hippo or other pathways, has been linked to the regulation of organ size, cell growth and differentiation during development, tissue regeneration, and cancer (6,7). In the liver, ectopic expression of YAP leads to hepatomegaly, associated with widespread hepatocytes dedifferentiation and cellular proliferation (8–10). Similarly, loss of function mutations of upstream inhibitors like MSTs and NF2 promote post-natal liver growth and tumorigenesis (11–15). Lineage tracing experiments indicated that YAP could reprogram adult hepatocytes to a bifunctional progenitor-like state, supporting differentiation along the hepatocyte or the cholangiocyte fate (10). The ability to reprogram somatic cells to a less differentiated multipotent state has also been confirmed in other tissues and appears to be one of the hallmark activities of YAP (16). Prolonged expression or activation of YAP in the liver promotes the emergence of tumoral lesions with features of either hepatocellular carcinoma or cholangiocarcinomas, thus demonstrating the oncogenic potential of YAP (8,9,11–15). In line with this, YAP can be found overexpressed and activated in liver tumors of human origin (11,14,17–19) and, more in general, in several solid tumors of different tissues (20,21). In particular, in aggressive and poorly differentiated tumor sub-types, besides controlling proliferation and promoting cell survival, YAP activity has been linked to EMT (22,23), acquisition of stem cell properties (20), cell migration (24), and chemoresistance (25,26).

Loss of YAP expression has mild effects on hepatocytes during homeostasis (12,27,28), yet YAP or YAP/TAZ null livers are severely impaired in regeneration following cholestatic injury (29,30). While these reports suggest a cell-autonomous role of YAP/TAZ in the regeneration of hepatocytes, recent data indicates that the major role for YAP/TAZ during liver regeneration is to preserve biliary epithelial cells and prevent anti-regenerative cholestasis (31). Despite the growing relevance of YAP in physiology and pathology and the considerable efforts made to elucidate the molecular details of its regulation, how YAP-dependent transcription supports cell growth and cell identity programs in the liver is still poorly understood. Here we report our effort to describe, at the genome-wide level, how YAP reshapes the transcriptional and epigenetic landscape in hepatocytes, thus controlling their proliferation and differentiation.

## MATERIALS AND METHODS

### Mice strains

For liver-specific transgene expression, Tet-YAP mice (Col1A1-YAP^S127A^ transgenic mice) (32) were crossed with LAP-tTA mice expressing the tTA tetracycline transactivator under the control of the LAP promoter (B6.Cg-Tg(tTALap)5Bjd/J; stock #003563, Jackson Laboratories). To suppress transgene expression, LAP-tTA transgenic mice were kept under continuous administration of food supplemented with doxycycline (625 mg/kg). For activation, four weeks old mice were subjected to a regular diet.

For short-term activation studies, tet-YAP mice were crossed with Alb-CRE mice (B6.Cg-Tg(Alb-cre)21Mgn/J, stock #003574, Jackson Laboratories) and ROSA26-rtTA-IRES-EGFP, rtTAflox mice (B6.Cg-Gt(ROSA)26Sortm1(rtTA,EGFP)Nagy/J; stock #005670 Jackson Laboratories). Mice were fed with doxycycline-supplemented food for up to 7 days. Animal experiments were performed following the Italian law (D.L.vo 116/92 and following additions) as approved by OPBA and authorized by the Ministry of Health.

### Antibodies

ChIP: anti-H3K27ac (Abcam, ab4729), anti-H3K4me3 (Active Motif, #39159), anti-H3K4me1 (Abcam, ab8895), anti-H3K122Ac (Abcam ab33309), anti-MycN262 (Santa Cruz, sc-764), anti-RNAPol2 N20-X (Santa Cruz, sc-899), anti-YAP1 63.7 (Santa Cruz, sc-101199), anti-TEAD4 (Aviva Systems Biology, ARP38276), anti-BRD4 (Bethyl, A301-985A100), anti-HNF4a (Abcam, ab41898). Normal rabbit/mouse IgG (Santa Cruz, sc-2027) was used as background control. Please note that the anti-TEAD4 (Aviva Systems Biology, ARP38276) was reported to recognize also TEAD1 and TEAD3 (33). Western-Blot: anti-YAP1 63.7 (Santa Cruz, sc 101199) and anti-TEAD4 (Santa Cruz, sc-101184); anti-HNF4a (Abcam, ab199431), anti-total H3 (Abcam, ab1791); anti-vinculin clone H (SigmaAldrich, V9131); anti-TEAD1 (Cell Signalling, #8526). Goat anti-rabbit HRP (Biorad, #1706515) and Goat anti-mouse HRP (Biorad, #1706516) were used as secondary antibodies. Immunohistochemistry: anti-YAP1 (Cell Signalling, #4911), anti-Ki67 (Thermo Scientific, #9106), anti-Sox9 (Millipore, #5535), anti-HNF4a (Santa Cruz, sc-8987 and Abcam, ab41898).

### Chromatin immunoprecipitation

For TEAD4, Histone Marks (H3K27Ac, H3K4me3, H3K4me1, H3K122Ac), HNF4a, BRD4, and RNAPol2 ChIP, dissected livers were fixed with 1% formaldehyde in PBS and quenched with 0.125 M of Glycine. For YAP ChIP, cells were fixed with 0.5 M DSG (di-(*N*-succinimidyl)-glutarate) for 45 min and then 1% formaldehyde (FA) in PBS 12 min. Fixed tissues were further processed as previously described (32). For sequencing purposes, 2–5 ng of ChIPed DNA was prepared for HiSeq2000/Novaseq 6000 sequencing with TruSeq ChIP sample preparation kit (Illumina) following the manufacturer's instruction.

### Assay for transposase accessible chromatin (ATAC-seq)

ATAC-seq was performed on 100 000 cells obtained from liver perfused mice (34). For library preparation, primers are listed in Supplementary Table S1. Quality control of the libraries was performed with Agilent 2100 Bioanalyzer (Agilent High Sensitivity DNA chip, #5067-4626). Library concentration was assessed by Qubit. Obtained ATAC-seq libraries were sequenced on the Illumina NovaSeq 6000 with 50bp paired-end. Cloning and qPCR primers are listed in Supplementary Tables S2 and S3.

## RESULTS

### Identification of YAP target sites in the adult liver

**Figure 1.**
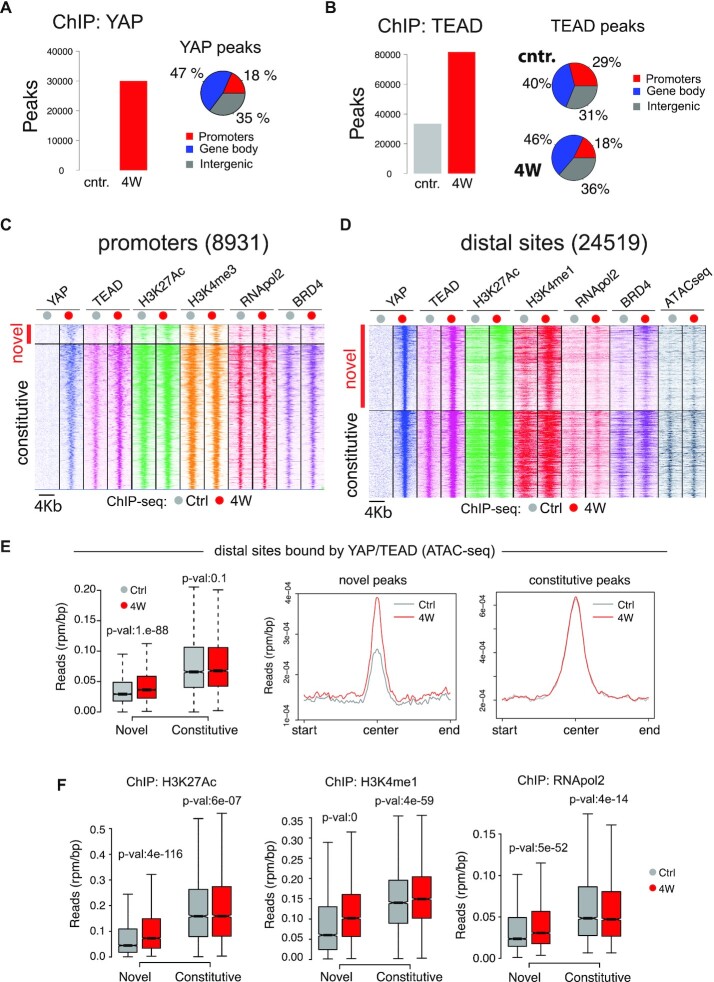
Chromatin association studies in LaptTA/tet-YAP^S127A^ livers upon YAP expression for four weeks (4W). Not induced littermates were used as controls (cntr.). (**A, B**) Peaks identified by ChIP-seq (bar-plot, left) and their distribution relative to coding genes (pie chart, right). (**C, D**) Heatmaps of ChIP-seq signals at the YAP/TEAD bound regions. (**E**) Analysis of nucleosome free-regions by ATAC-seq at novel and constitutive YAP/TEAD bound sites. Left: box-plot of nucleosome-free signals. Right: nucleosome-free signals. (**F**) ChIP-seq signals at distal sites bound by YAP/TEAD.

To address how YAP controls transcription in adult hepatocytes, we analyzed the liver of LaptTA/tet-YAP^S127A^ transgenic mice. YAP^S127A^ is a constitutively active protein, point-mutated in one of the inhibitory phosphorylation sites. As reported (8,9), its expression led to proliferation and dedifferentiation of hepatocytes, hepatomegaly, and liver tumors (32). We conducted ChIP-seq analyses at 4 weeks of YAP^S127A^ induction, when hepatomegaly, cell dedifferentiation, and proliferation are fully established (32). YAP^S127A^ showed broad genome-wide binding, with ∼30 000 peaks, prevalently at distal regions (Figure 1A). Few YAP peaks were detected in wild-type livers, while TEAD, the main partner of YAP, showed widespread genome binding already in wild-type conditions, with ∼30 000 peaks detected, mainly at distal regions (Figure 1B). Analysis of chromatin modifications suggested that most of the distal TEAD bound loci were enhancers (Supplementary Figure S1A, B). Induction of YAP^S127A^ increased TEAD binding, resulting in 80 000 peaks detected (Figure 1B). This increase was partly due to YAP-dependent positive feedback leading to the upregulation of TEAD levels (see below, Figure 6 and related text). Comparative analysis revealed strong co-occurrence of YAP and TEAD on two classes of TEAD bound sites (Supplementary Figure S1B–D). The first class comprised sites constitutively bound by TEAD: of these, half were bound by YAP with high affinity (Supplementary Figure S1B, C). YAP binding to these constitutive sites increased TEAD enrichment, which was stronger than that observed at sites bound only by TEAD (Supplementary Figure S1E). This suggests that the YAP/TEAD heterodimer displays an increased affinity for its genomic targets than TEAD-only complexes. The second class of TEAD-bound loci, more prevalent at distal sites, included loci bound by YAP/TEAD only upon YAP induction (i.e. novel YAP/TEAD sites, Figure 1C, D). On these YAP/TEAD sites, YAP binding was associated with increased chromatin accessibility (Figure 1E), higher deposition of enhancer-associated activatory chromatin marks (i.e. H3K4me1, H3K27ac), and stronger RNApol2 recruitment (Figure 1F). Overall, this suggested a pioneer activity of YAP/TEAD dimers, which promotes activation of enhancers, possibly by recruiting chromatin-remodeling and histone-modifying complexes.

### Genome-wide expression analysis following YAP^S127A^ induction in the liver

**Figure 2.**
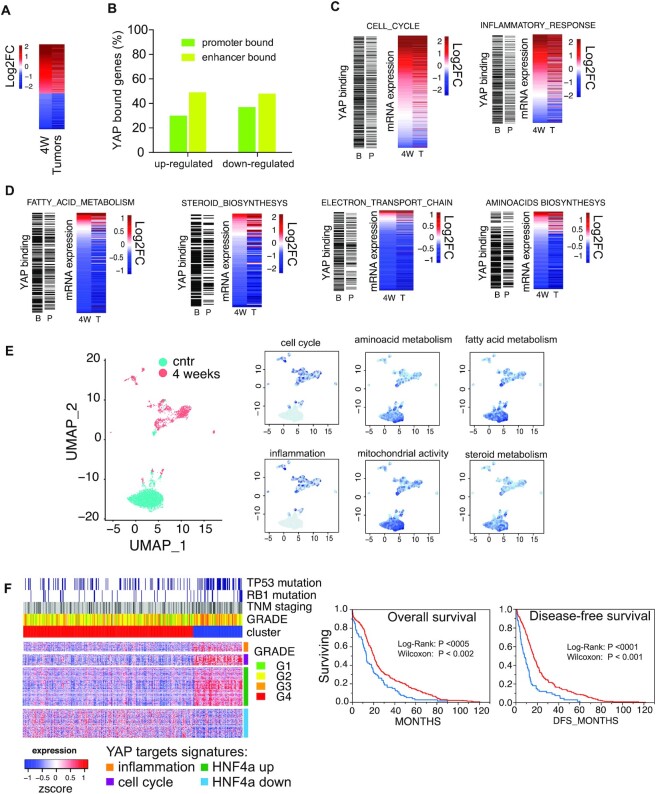
Expression analysis of LaptTA/tet-YAP^S127A^ livers upon YAP induction for four weeks (4W). (**A**) Heatmap of the log_2_ fold change of the differentially expressed genes identified upon four weeks of YAP induction or in YAP-induced tumors by RNA-seq. (**B**) Fraction of the up- or down-regulated genes bound by YAP either at their promoter or enhancer. (**C, D**) Summary of GSEA analysis of the pathways and processes (i.e. gene signatures) regulated by YAP. For each group of signatures, the expression changes (log_2_FC) and YAP binding at either promoters (P) or promoters and proximal enhancers (B = both) are reported. (**E**) scRNA-seq analysis of hepatocytes. Left: UMAP of hepatocytes. Right: UMAPs color-coded for the intensity of the enrichment of selected transcriptional signatures. (**F**) Left: clustered Heatmap of HCCs from TCGA, right: Kaplan–Meier plot of the overall and the disease-free survival data for the two clusters (blue: high YAP signature, red: low YAP signature).

RNA-seq analysis identified ≈4500 genes significantly deregulated by YAP^S127A^: 2538 genes were upregulated, while 1851 were downregulated (Figure 2A). These genes were also deregulated in liver tumors, which developed upon prolonged induction of YAP^S127A^ (Figure 2A and Supplementary Table S4). A large fraction of regulated genes were bound by YAP or proximal to a YAP-bound enhancer (Figure 2B, C, and Supplementary Figure S3). GSEA analysis indicated that the upregulated genes were enriched in pathways related to cell cycle progression, cellular proliferation, and mitogenic signaling (Figure 2C; Supplementary Figures S2 and S3; Supplementary Table S5). Upregulated genes were also enriched in inflammatory genes, particularly immunomodulatory cytokines and chemokines (Figure 2C and Supplementary Figures S2 and S3); and scRNA-seq profiling confirmed that the above-identified immunological signatures were enriched in hepatocytes (Figure 2E, Supplementary Table S6). Hepatocytes from YAP-activated mice exhibited strong positive enrichment for IFNλ signaling and TNFa signaling via NF-kB (Supplementary Figure S4), with almost 30% of all genes analyzed that were NF-κB targets (35) (Supplementary Table S7). Several immunomodulatory cytokines and chemokines, along with some receptors, were induced upon YAP overexpression (36) (Supplementary Table S8). Among them, we confirmed the upregulation of Ccl2 and Csf1, which were previously shown to be required for the recruitment of tissue infiltrating macrophages during YAP-induced liver carcinogenesis (36).

Downregulated genes were enriched for metabolic signatures related to amino acid, steroid, fatty acid biosynthesis, and mitochondrial energy production, as well as RNA metabolism (Figure 2D and Supplementary Figures S2 and S3). scRNA-seq analysis confirmed that these pathways were deregulated in hepatocytes (Figure 2E). Closer inspection of the genes involved in cholesterol metabolism revealed that while there was a general downregulation of biosynthetic genes, there was also upregulation of genes involved in the uptake and its metabolic conversion, thus suggesting that YAP may lead to the repression of *de novo* cholesterol synthesis and a compensatory activation of cholesterol uptake and its use as a metabolic intermediate available for hormones and bile acid synthesis (Supplementary Figure S5). A large fraction of down-regulated genes were targets of HNF4a (Figure 4A), a transcription factor that orchestrates differentiation of the hepatic lineage (37). To evaluate whether the identified YAP-dependent transcriptional programs were altered in human HCCs, we derived a signature of YAP targets genes (i.e. bound and regulated, Supplementary Table S9), which was used to cluster tumors from the TCGA human liver cancer dataset. *K*-means clustering highlighted a subset of high-grade liver tumors enriched for TP53 and RB1 mutations (Figure 2F). Liver cancer patients stratified by this signature showed a worse prognosis, both in overall and in disease-free survival (Figure 2F). This confirmed that YAP-regulated genes are associated with aggressive tumor growth and suggested a crucial role for YAP in controlling and determining their transformed state.

### Transcriptional activation at YAP target genes

**Figure 3.**
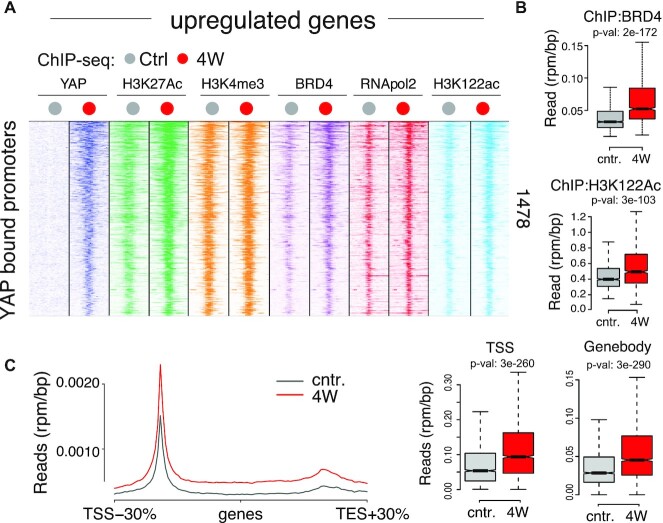
Transcriptional activation by YAP in LaptTA/tet-YAP^S127A^ livers upon YAP expression for four weeks (4W). (**A**) Heatmap of ChIP-seq signals at promoters of genes upregulated and bound by YAP (851 genes). (**B**) ChIP-seq signals at the promoters shown in (A). (**C**) Left: distribution of RNApol2 ChIP-seq signals along genes upregulated and bound by YAP. Right: box-plots of RNApol2 levels at transcription start sites (TSS) and gene bodies (GB).

To gain insight into the mechanism of gene activation, we analyzed the promoters of genes bound and upregulated by YAP. These genes were lowly expressed in wild-type livers (Supplementary Figure S6), were pre-marked by activatory chromatin marks such as H3K27Ac and H3K4me3, and displayed a low level of promoter-bound RNApol2 (Figure 3A and Supplementary Figure S7). YAP binding was associated with the recruitment of BRD4 and a sharp increase in H3K122Ac, which in turn favored both the recruitment of RNApol2 and its release from promoters (Figure 3B and C). This is in agreement with previous data showing that YAP regulates RNApol2 pausing (38) and that in cancer cells, YAP-dependent transcription relies on BRD4 activity (39). Similar changes, albeit with lower intensities, were also noticeable on the promoters of genes bound but not upregulated by YAP. Here, YAP binding was associated with a further increase in BRD4 and concomitant elevation of H3K122Ac; yet these events did not associate with an increase in RNAPol2 recruitment and activity, conceivably because these genes were already transcribed at high levels (Supplementary Figures S6 and S7) (39). Interestingly, there was a progressive increase in gene activation when comparing promoter bound genes to those associated with constitutive enhancers or novel enhancers bound by YAP, underscoring how the engagement of distal elements by YAP led to robust transcriptional activation (Supplementary Figure S7B).

### YAP represses genes activated by HNF4a

**Figure 4.**
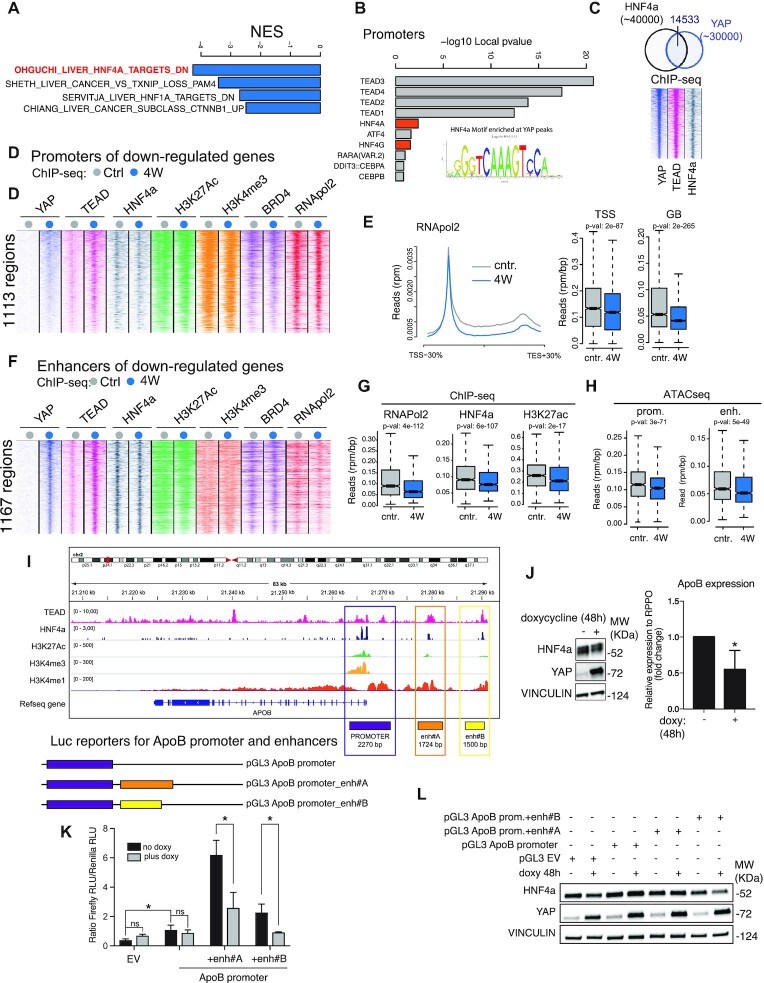
YAP antagonizes HNF4a activity. (**A**) GSEA of YAP down-regulated genes (top four signatures). (**B**) Transcription factor binding motifs at YAP bound promoters. Inset: HNF4a binding motif. (**C**) Top: overlap of YAP and HNF4a ChIP-seq peaks identified in liver cells. Bottom: Heatmap of the ChIP-seq signals. (**D**) Heatmap of the ChIP-seq signals found at promoters. (**E**) Metagene plot of RNApol2 ChIP-seq signals (left) and box plot of RNApol2 ChIP-seq signals (right) at transcription start sites (TSS) and along gene bodies (GB) for the gene shown in (D). (**F**) Heatmap of the ChIP-seq signals at HNF4a/YAP bound enhancers proximal to genes down-regulated by YAP. (**G**) ChIP-seq signals at the enhancers shown in (F). (**H**) Nucleosome-free regions at the promoters and the enhancers shown in (D) and (F). (**I**) Genomic snapshot of the APOB locus. The promoter region and the putative enhancers are outlined. (**J**) Left: western-blot analysis of HNF4a and YAP levels in tet-YAP HEPG2 cells where YAP^S127A^ was induced for 48 h. Vinculin was used as an internal standard. Right: APOB mRNA expression analysis by RT-qPCR. Average and standard deviation of three independent experiments is shown. (**K**) Luciferase based reporters used and bar plot of the luciferase assay performed in tet- YAP^S127A^ HEPG2 cells. The average and standard deviation of three independent experiments is shown. (**L**) Analysis of YAP and HNF4a levels by Western-blotting. Vinculin was used as loading control.

GSEA on the most down-regulated transcripts revealed that HNF4a target genes were predominantly repressed by YAP (Figure 4A), in accordance with previous reports (40–42).

HNF4a (hepatocyte nuclear factor 4 alfa) is a transcription factor (TF) of the nuclear receptor family which controls the expression of gene programs essential for hepatocyte differentiation during embryonic development and in the adult liver (37).

Downregulation of HNF4a responsive genes did not associate with decreased HNF4a levels, nor it depended on genome-wide loss of HNF4a binding (Supplementary Figure S8A–C). This prompted us to investigate how YAP controlled the expression of HNF4a target genes. TF binding motif analysis identified the HNF4a motif as significantly enriched at YAP target sites (Figure 4B and Supplementary Figure S8D), and ChIP-seq showed genome-wide co-occurrence of YAP/TEAD and HNF4a (Figure 4C). This co-occurrence was prominent on promoters of YAP-downregulated genes, which were constitutively bound by HNF4a and TEAD, and then by YAP upon its induction (Figure 4D). The genome-wide proximity of YAP/TEAD and HNF4a could also be confirmed in the HEPG2 hepatocellular carcinoma cell line: analysis of the publicly available ChIP-seq data (encode database) showed strong co-localization of TEAD and HNF4a (Supplementary Figure S9A). This analysis also showed the genomic proximity of TEAD with other TFs predicted to co-occur based on our TF binding motif analysis (e.g. RXR, HNFg) and revealed TEAD co-occurrence with TFs regulating metabolic genes (e.g. USF1) and with FOXA1, a pioneer transcription factor recently shown to participate in YAP mediated gene regulation (43) (Supplementary Figure S9).

Expression of YAP^S127A^ in HEPG2 cells led to the repression of genes enriched for signatures of liver cancer genes and HNF1,4a activated genes (Supplementary Figure S9D, Supplementary Table S10). We also identified HNF4a activated genes in HEPG2 cells by performing RNA-seq analyses upon knock-down of HNF4a (Supplementary Table S11): 75% of these genes were downregulated by YAP^S127A^, thus confirming the antagonism of YAP and HNF4a on genes upregulated by HNF4a (Supplementary Figure S9E, Supplementary Table S12).

In mouse liver, while upon YAP binding, there was a sharp increase of TEAD enrichment, only a negligible variation of HNF4a binding was observed, thus ruling out competition between YAP/TEAD and HNF4a for promoter binding (Figure 4D and Supplementary Figure S10A). The levels of promoter-associated RNApol2 and activatory chromatin marks were marginally affected by YAP binding, thus indicating that YAP did not alter epigenetic priming nor RNApol2 recruitment (Figure 4D and Supplementary Figure S10A, see also Figure 4E for RNApol2 binding at TSS). Gene-body associated RNApol2 was dramatically reduced, coherently with the down-modulation of transcription of these HNF4a targets (Figure 4E). Given the prominent role of enhancers in regulating RNApol2 pause-release and considering both the preferential binding of YAP/TEAD to distal regulatory regions (44,45) and the role of HNF4a in activating liver enhancers (46), we analyzed enhancers associated with down-regulated genes: 37% of the promoters of downregulated genes were proximal to an enhancer bound by both YAP and HNF4a (Figure 4F and Supplementary Figure S10B, D). These enhancers showed strong YAP-dependent recruitment of TEAD, which was associated with a reduction in RNApol2, HNF4a, and the activatory mark H3K27ac (Figure 4F, G and Supplementary Figure S10C). These events were linked to decreased chromatin accessibility, both at enhancers and promoters (Figure 4H), suggesting that YAP binding disrupted promoter-enhancer contacts and altered their topology, thus impairing RNApol2 pause-release on HNF4a target genes. This pointed to a significant role of enhancers in controlling the activity of HNF4a target genes. Seeking direct evidence, we focused on the canonical HNF4a target ApoB, which, in HEPG2 cells, is bound by TEAD and HNF4a, both at the promoter and at two proximal putative enhancers (Figure 4I). As expected, the expression of YAP^S127A^ led to the repression of ApoB (Figure 4J). We thus devised a set of luciferase-based reporter genes containing the ApoB promoter, either alone or in combination with the proximal enhancers, and probed their constitutive activity and their modulation upon YAP^S127A^ expression (Figure 4K). While the promoter alone showed low luciferase activity, its pairing to either of the two enhancers potentiated the reporter activity, thus indicating the functional relevance of these proximal enhancers in the regulation of ApoB (Figure 4L). Conditional expression of YAP^S127A^ repressed the luciferase activity, most prominently when the reporter genes included the enhancers, thus indicating that, indeed, YAP was particularly effective in abating enhancer mediated gene expression (Figure 4K).

### YAP regulates developmental enhancers to antagonize lineage specification

**Figure 5.**
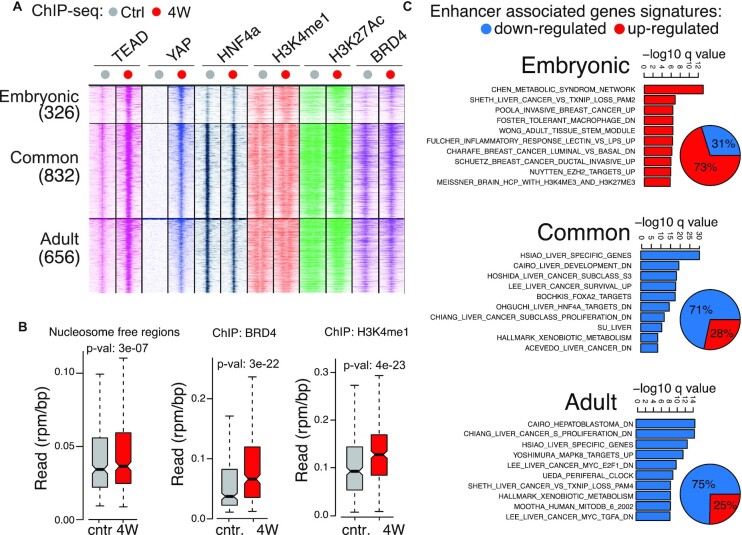
Developmental enhancers are regulated by YAP. The analysis was performed in LaptTA/tet-YAP livers upon YAP expression for four weeks (4W). (**A**) Heatmap of the ChIP-seq signals at developmental enhancers bound by YAP. (**B**) ATAC-seq (nucleosome free regions) and ChIP-seq signals at embryonic enhancers. (**C**) GSEA analysis of the genes associated to common, adult or embryonic enhancers. Left: pie chart of upregulated and downregulated genes for each class of enhancers.

In the liver, lineage specification and cellular identity are regulated by master TFs like HNF4a and FOXA2, which control transcriptional programs both in the embryonic liver and postnatally by engaging different classes of enhancers: embryonic, adult, and constitutive enhancers (40). We evaluated whether the activation of YAP in the adult liver would affect either class of enhancers. A consistent number of common and adult enhancers were constitutively bound by TEAD and, following its induction, also by YAP^S127A^ (Figure 5A, Supplementary Figure S11A and Supplementary Table S13). Consistent with the antagonistic action of YAP and HNF4a (40–42), these enhancers were preferentially associated with genes involved in liver function/identity, which were downregulated by YAP (Figure 5B). A fraction of embryonic enhancers (30%) was re-engaged by YAP, which promoted TEAD binding and their activation, as evidenced by increased accessibility, H3K4me1, and BRD4 binding (Figure 5A, B, Supplementary Figure S11B, C and Supplementary Table S13). These enhancers were associated with genes linked to cell motility, mesenchymal/stem cell phenotypes, and inflammation (Figure 5C). Overall, this suggests that YAP triggers the epigenetic remodeling of the enhancer landscape, which leads to the repression of lineage specification programs and the (re)-activation of embryonic programs.

### TEAD dependent feed-forward loop supports tonic YAP activity

**Figure 6.**
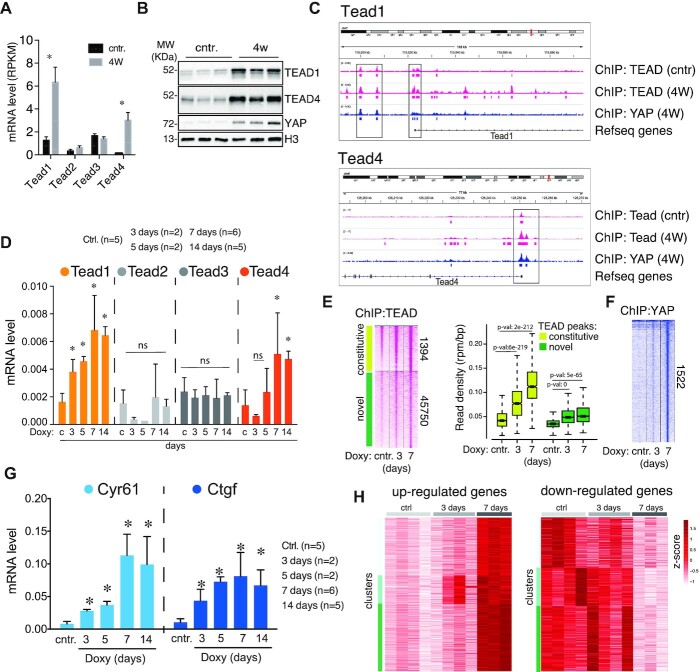
YAP induces a feed-forward loop based on transcriptional regulation of Tead1 and Tead4. (**A**) Tead's expression in LaptTA/tet-YAP^S127A^ livers by RNA-seq. Bar-plot shows the average and standard deviation of three independent experiments. (**B**) Western-blotting analysis of TEADs in LaptTA/tet-YAP^S127A^ livers. (**C**) Genomic snapshot of the Tead1 and Tead4 loci annotated with the ChIP-seq tracks for TEAD and YAP. (**D**) Evaluation of Teads mRNA by RT-qPCR analysis upon YAP induction in R26-lsl-rtTA/alb-CRE/tet-YAP^S127A^ mice. *N* = number of independent livers. Doxy = doxycycline. (**E**) Heatmap (left) and box-plot (right) of ChIP-seq signals of R26-lsl-rtTA/alb-CRE/tet-YAP^S127A^ liver cells. YAP was induced for the indicated days. (**F**) Heatmap of YAP ChIP-seq signals of R26-lsl-rtTA/alb-CRE/tet-YAP^S127A^ liver cells, upon YAP induction for the indicated days. (**G**) RT-qPCR analysis of liver cells from R26-lsl-rtTA/alb-CRE/tet-YAP^S127A^ mice. (**H**) Heatmap of normalized gene expression by RNA-seq upon YAP induction in R26-lsl-rtTA/alb-CRE/tet-YAP^S127A^ mice. Left: genes upregulated by YAP; right: genes down-regulated by YAP. *P value <0.01 (*t*-test).

ChIP-seq analyses indicated an increase in TEAD binding following YAP^S127A^ induction (Figure 1B). Evaluation of TEADs levels upon YAP activation revealed a rise in TEAD1 and 4, both at the mRNA and protein levels (Figure 6A and B). ChIP-seq showed binding of YAP and TEAD at the promoters of these genes and also at proximal enhancers (Figure 6C). This suggested the existence of a positive feed-forward loop. Short-term expression analysis in R26-lsl-rtTA/alb-CRE/tet-YAP^S127A^ revealed that TEAD1,4 levels raised progressively to reach a plateau of maximal expression at seven days of YAP induction (Figure 6D, Supplementary Figure S12). This was associated with a boost in YAP/TEAD chromatin binding (Figure 6E, F). YAP level was already maximal at three days, thus suggesting that increased availability of TEADs favored chromatin binding by the YAP/TEAD heterodimers (Supplementary Figure S12). This progressive increase in YAP/TEAD genome binding matched the gradual rise of expression of YAP canonical target genes and, more broadly, the boost in transcription in the tonic phase of YAP activation (7 days) (Figure 6G, H and Supplementary Table S14). Significantly, high YAP-dependent transcription was associated with a robust proliferation, dedifferentiation of hepatocytes, acquisition of cholangiocyte markers, and broad alteration(s) of the liver parenchyma (Supplementary Figure S12B, C). We propose that this feed-forward regulation may represent an inherent requirement for the tonic activation of YAP, which is needed to support undifferentiated progenitors during embryonic development or somatic regeneration and, in pathological settings, to ensure the viability and growth of cancer cells.

### YAP induction results in remodeling of the cellular microenvironment

**Figure 7.**
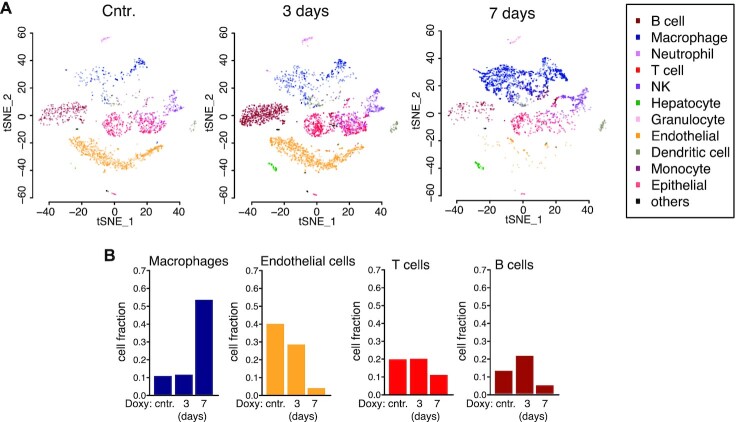
Single cell profiling of the liver microenvironment of R26-lsl-rtTA/alb-CRE/tet-YAP^S127A^ mice. (**A**) t-SNE classification and in-silico cell type annotation. (**B**) Relative cell numbers of the most relevant cell types.

The profound transcriptional and histological changes observed in the liver upon tonic YAP induction led us to evaluate changes in cellular heterogeneity by scRNA-seq. t-SNE identified 11 cell types (47) (Figure 7A). Predominant groups were cells of the immune system (T-cells, B-cells and macrophages) and endothelial cells. While acute activation had little effects, tonic YAP-induction led to a fractional decline of T-lymphocytes, B-lymphocytes, and endothelial cells with a concomitant rise in the fractional abundance of macrophages (Figure 7B and Supplementary Figure S13). Coherently, the major transcriptional changes were observed after tonic YAP induction (7 days) (Supplementary Tables S15–S17). Macrophages displayed features of M2 polarization, such as the downregulation of MHC class I and II components and the increase in the expression of both TGFb and genes linked to the oxidative metabolism (Supplementary Figure S14). They also showed upregulation of genes linked to cellular motility, chemotaxis, and recruitment of immune cells (Supplementary Figure S14C, E). T-lymphocytes displayed the downregulation of cytotoxic programs, likely in response to the immunomodulatory action of macrophages (Supplementary Figure S15). Interestingly, the endothelial cells observed on day 7 had prominent proliferative gene signatures, possibly reflecting an angiogenic-remodeling of the vascular microenvironment (Supplementary Figure S16).

## DISCUSSION

Our analysis confirms that YAP binding to its genomic targets is invariably associated with TEAD (44,45). We identified two broad classes of YAP-bound genomic loci: (i) constitutive YAP/TEAD sites, which were bound by TEAD in a YAP independent way and (ii) novel YAP/TEAD sites, which TEAD did not bind in the absence of YAP. One major determinant for the affinity of YAP to the constitutive YAP/TEAD sites appeared to be TEAD enrichment, as indicated by the evidence that the YAP-bound constitutive sites had higher TEAD enrichment than TEAD-only sites (Supplementary Figure S1B, C and E). In addition, these sites were generally more enriched in chromatin modification and binding of both RNApol2 and BRD4, suggesting that chromatin-modifying complexes and transcriptional activators may also contribute to increasing TEAD and YAP/TEAD affinity for these target loci. On the other hand, novel YAP/TEAD sites were prevalently poised/inactive enhancers: YAP/TEAD binding coincided with a gain in chromatin accessibility, increase in H3K4me1, H3K27Ac and recruitment of BRD4 and RNApol2, thus implying that chromatin independent assembly of YAP/TEAD heterodimers, and their ability to recruit chromatin remodeling complexes (and possibly pioneer factors), are critical events for transcriptional regulation at these loci. This resonates with recent data showing that conditional expression of activated YAP in adult cardiomyocytes leads to chromatin remodeling and increased accessibility that triggers enhancer-mediated transcription of fetal-like genes (48). We have identified promoters that are bound and activated by YAP. Our data confirm the role of YAP in promoting the recruitment of RNApol2 in a BRD4-dependent way and its role in controlling elongation in cancer cells (38,39). It also indicates that the BRD4-dependent regulation of YAP target genes is not a prerogative of cancer cells but a more general mechanism used by YAP to regulate gene expression also in normal cells. This raises the question of the selectivity and efficacy of BRD4 inhibitors in targeting YAP-dependent transcriptional addiction in cancer cells. We note that contrary to what is observed in cancer cells, where YAP targets are highly expressed genes (39), YAP targets are expressed at normal levels in hepatocytes. Thus, transcriptional addiction to YAP might be due to the transcriptional amplification of its targets in cancer cells. Interestingly, in tumors cells, YAP targets are enriched in Myc regulated genes (32), possibly suggesting that co-activation by YAP and Myc, while potentiating gene expression, may also render cancer cells liable to BET inhibitors and more in general to transcriptional inhibitors.

We also detected a large number of genes that are repressed by YAP: these genes control liver cells metabolism and are regulated by HNF4a, a master regulator of lineage specification and cell function in the liver (49). Transcriptional antagonism between YAP and HNF4a has been reported (40–42,50), but a complete understanding of its molecular details is missing. Our data provide further mechanistic insight and indicate a direct role of YAP, which, by binding to HNF4a occupied loci, both promoters, and their cognate enhancers, impairs RNApol2 promoter escape. This peculiar repressive mechanism is reminiscent of how YAP antagonizes SMAD-dependent gene activation by reinforcing NELF mediated RNApol2 stalling, thus impeding differentiation of embryonic stem cells towards a mesenchymal fate (51). We propose that the enhancer-mediated control of RNApol2 promoter escape may be a general repressive mechanism that YAP uses to prevent or revert lineage differentiation. In the liver, YAP repressed promoters and their associated enhancers are constitutively bound by TEAD and HNF4a. The coexistence of antagonistic transcription factors and the resilience of stalled RNApol2 at their promoters upon YAP binding may be necessary to provide the cellular plasticity which characterizes adult hepatocytes and could be the molecular reason for both the swift dedifferentiation of hepatocytes upon YAP activation and the full reversibility of this process (8,10,41). HNF4a transcriptional control, both during liver development and postnatally, relies on its ability to temporally regulate the activity of embryonic and adult enhancers in a concerted manner with the pioneer transcription factors FOXA1,2 (40). HNF4a also provides steady activation of common liver enhancers, a class of liver-specific distal regulatory elements that are operative throughout liver development and adult life (40). TEAD has been previously linked to the control of the activity of the embryonic enhancers during development (40). Here, we expand on this and show that not only activation of YAP in the adult leads to the re-engagement of a fraction of embryonic enhancers and their cognate genes, but we also provide strong evidence for a pervasive antagonistic regulation by YAP/TEAD and HNF4a on the common and the adult enhancers, the other two prevalent classes of HNF4a regulated enhancers. This transcriptional antagonism between YAP and HNF4a may reflect a broader regulatory network orchestrating metabolic and cell identity gene programs in the liver, which may comprise TFs like RXR/LXR, SREBP1, and CEBPs, (Supplementary Figures S9 and S10).

Interestingly, RXR/LXRs are ligand-dependent activators of cholesterol and lipid biosynthesis, and repressors of pro-inflammatory genes (52). The fact that cholesterol *per se* has anti-inflammatory activity (52) suggests a relationship linking the metabolic state to the immune-modulation in the liver, which may be transcriptionally regulated by networks of transcription factors including YAP, HNF4a and RXR/LXR.

YAP activation in adult hepatocytes not only drives their dedifferentiation to a bi-potent progenitor-like state but also leads to profound alterations of the liver vasculature and the immune infiltrate. Hepatocytes likely control the remodeling of the immune microenvironment through the activation of TNFa and IFN-λ inflammatory programs and the engagement of NF-κB. Notably, several of these genes showed YAP binding at their regulatory regions (Figure 2D), thus suggesting direct transcriptional regulation. Many of these genes are pro-inflammatory and immunomodulatory cytokines and chemokines, such as Ccl2 and Csf1, which may promote the recruitment and differentiation of M2-like tissue infiltrating macrophages, thus favoring the eviction and the decommissioning of cytotoxic T-lymphocytes. Thus, by orchestrating paracrine signaling from hepatocytes, YAP may promote the establishment of an immune-tolerant environment and, therefore, sustain broad cellular remodeling of the liver. Interestingly, in skin carcinomas, the expression of IFN-λ regulated genes is also YAP dependent (53), thus suggesting that modulation of the inflammatory response might be a hallmark of YAP activation. Future work will be needed to understand the molecular details of their regulation and establish the relevance of these programs in regeneration and cancer. We identified TEAD1,4 as direct targets of YAP. Activation of this positive feed-forward regulatory loop marked the transition from an acute activation of YAP, which may be required for signal-regulated pathways, to the tonic activation of YAP, necessary for the full engagement of transcriptional programs controlling cell identity, proliferation, and metabolism as well as the paracrine mediated remodeling of the liver parenchyma. We propose that this feed-forward regulation might be an inherent requirement for the tonic activation of YAP, which is needed to support undifferentiated progenitors during embryonic development or somatic regeneration and, in pathological settings, to ensure the viability and growth of cancer cells.

## DATA AVAILABILITY

All NGS data of this study (raw and processed) were deposited in the gene expression omnibus (GEO) database (54) under the accession number GSE138191.

## Supplementary Material

gkac624_Supplemental_Files

## Notes

Present address: Elisa Donato, Division of Stem Cells and Cancer, German Cancer Research (DKFZ) and Heidelberg Institute for Stem Cell Technology and Experimental Medicine (HI-STEM gGmbH), 69120, Heidelberg, Germany.

Present address: Francesca Biagioni, Kiadis Pharma, Amsterdam, The Netherlands.

Present address: Arianna Sabò, QUANTRO Therapeutics GmbH, Vienna, Austria.

Present address: Andrea Bisso, Gadeta BV, Utrecht, The Netherlands.
