# Supplementary material for: Decoding YAP dependent transcription in the liver
Source: Nucleic Acids Res. 2022 Jul 25;50(14):7959–71. doi: 10.1093/nar/gkac624 (PMC9371928; doi:10.1093/nar/gkac624)
Supplement: gkac624_Supplemental_Files [file gkac624_supplemental_files.zip › supplementary figures_and_methods_revTXT.pdf]

Supplementary figures

Suppl figure 1

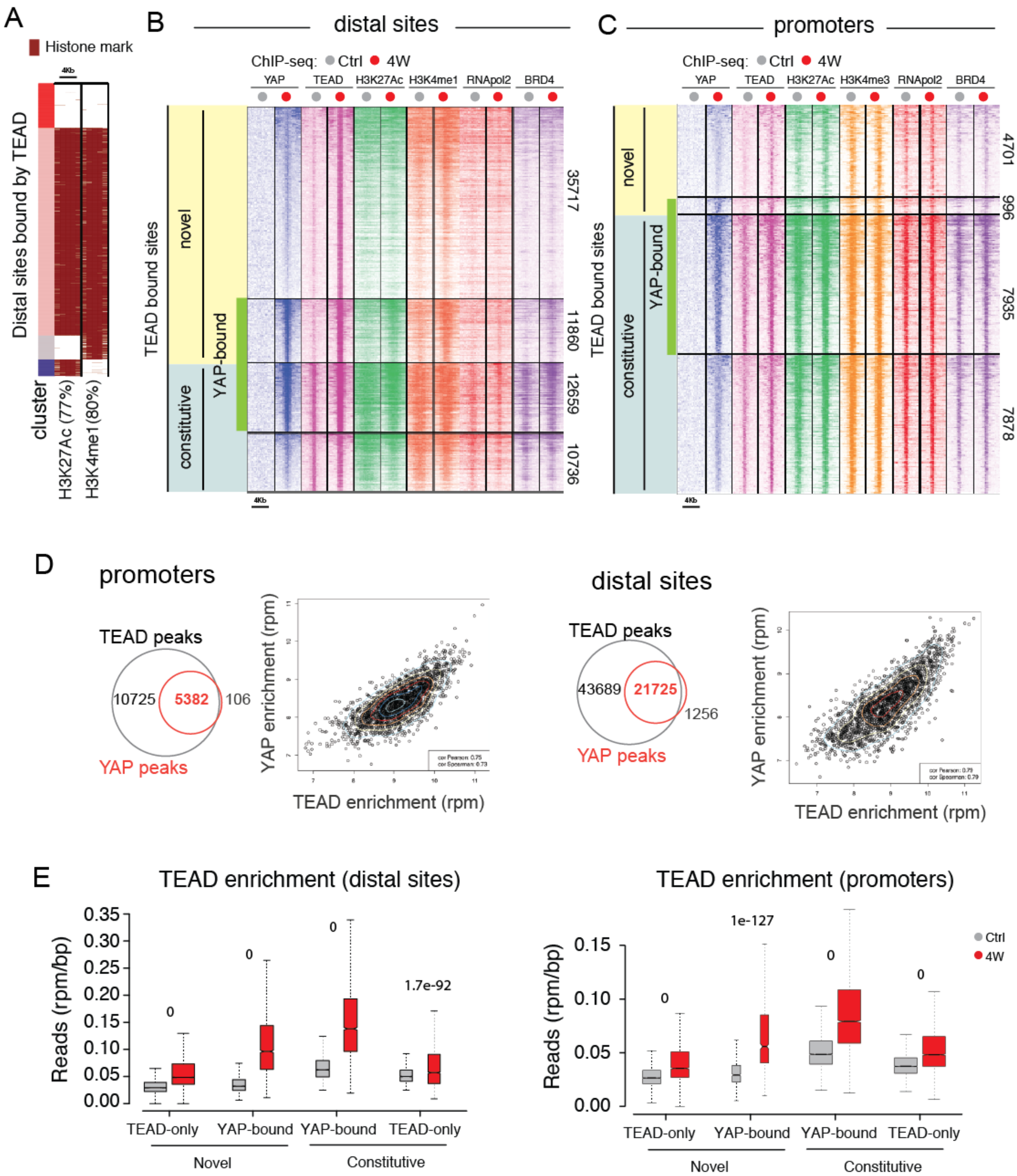

Figure legend is on the next page

Supplementary Figure 1. ChIP-seq analysis of wild-type (cntr.) and YAP expressing livers (4W= four weeks of YAP induction) from LaptTA/tet-YAP^{S127A} mice.
(**A**) Position-based clustered heatmap of H3K27ac and H3K4me1 ChIP-seq peaks mapped to distal regions bound by TEAD in wild-type liver. This analysis shows that the majority of TEAD bound distal sites are either active or poised enhancers. (**B, C**) Ranked heatmaps of the ChIP-seq signals at the genomic regions bound by TEAD. "YAP-bound" are TEAD bound regions overlapping with YAP peaks. (**D**)Co-occurrence of YAP and TEAD peaks upon YAP expression (Venn diagram, left) and their ChIP-seq signal intensities (right). (**E**) Box-plots of TEAD ChIP-seq signals on constitutive or novel sites, both on promoters and distal regions. Note that the enrichment of TEAD upon YAP expression increases more at YAP bound sites than at TEAD-only sites, thus suggesting that the YAP/TEAD complex has greater chromatin affinity compared to TEAD only. On the other hand, at the TEAD-only sites, there is a mild increase of TEAD upon YAP induction, which is driven by the increase in TEAD level supported by the feed-forward loop described in figure 6 and related text. Numbers in box-plots indicated the p-value.

Suppl figure 2

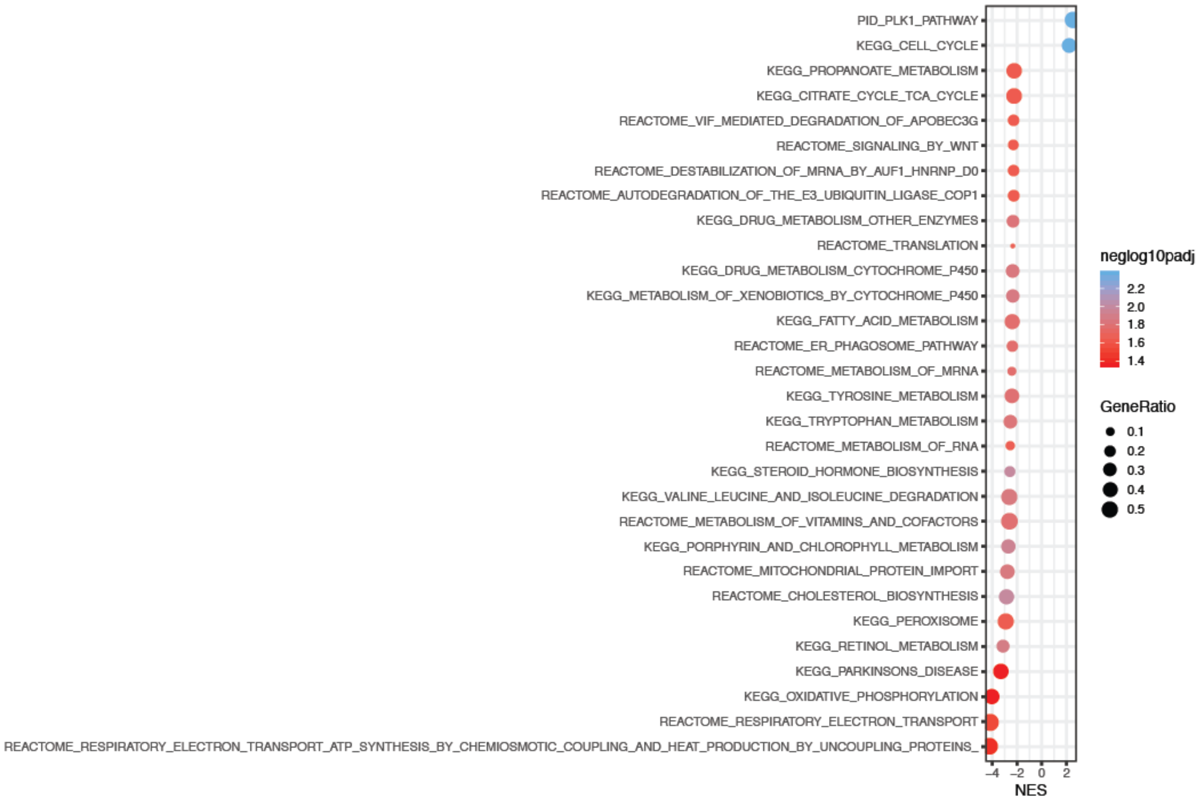

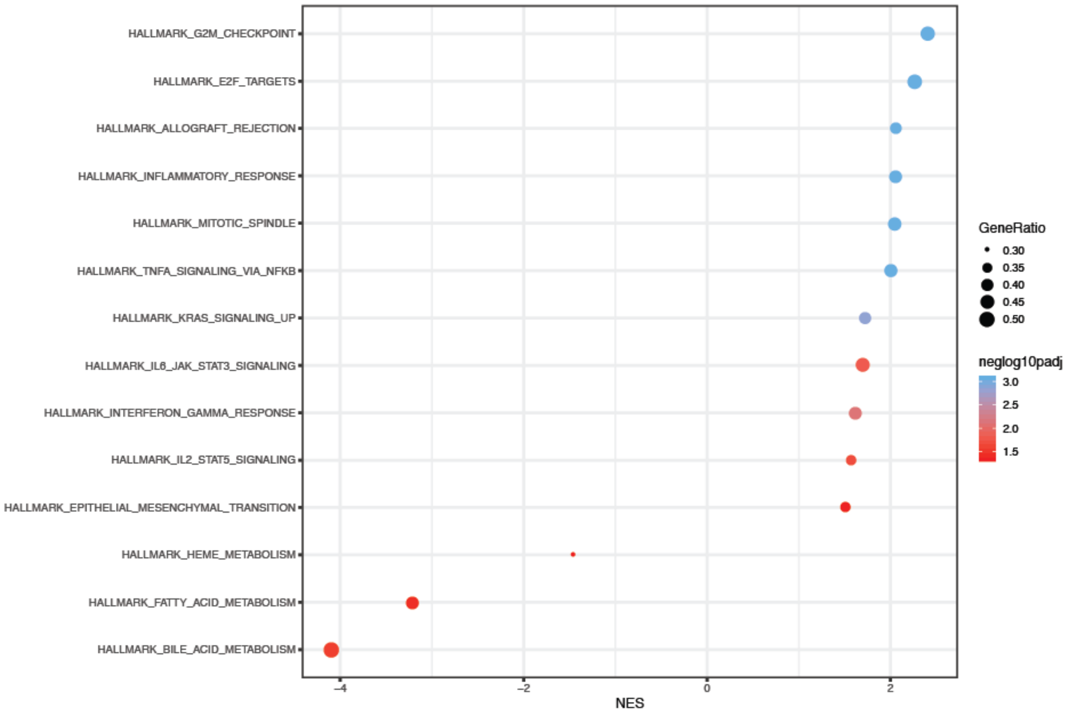

Supplementary Figure 2. GSEA analysis of the differentially expressed genes (DEGs) identified in LaptTA/tet-YAP^{S127A} liver following four weeks of YAP induction. Gene sets of MSigDB used for the analysis are C2 pathways (top panel) and Hallmarks (bottom panel). The normalized enrichment score (NES), -log10 p adjusted (neg. log10 padj) and the fraction of the genes are shown.

Suppl figure 3

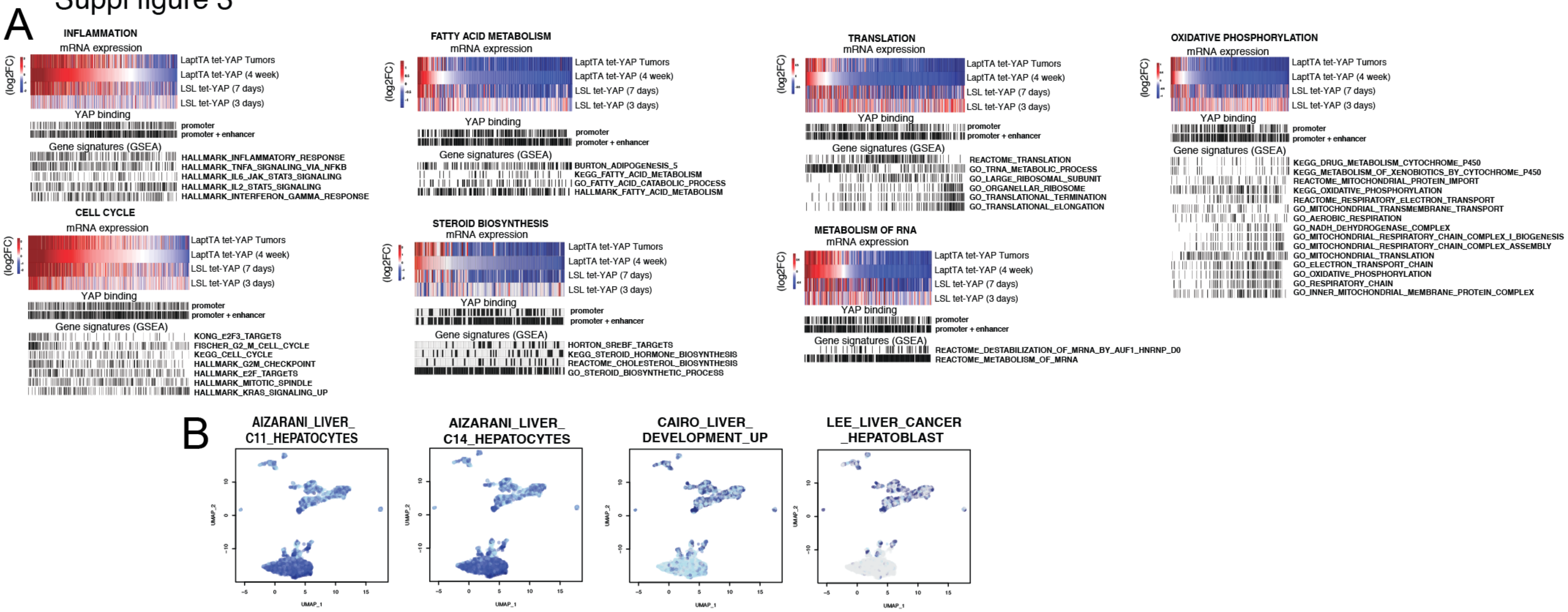

Supplementary Figure 3. (**A**) GSEA analysis of the differentially expressed genes (DEGs) identified in LaptTA/tet-YAP^{S127A} liver following four weeks of YAP induction. The “mRNA expression” heatmap shows the fold-change of the DEGs in the indicated mouse models: LaptTA/tet-YAP^{S127A} and R26-LSL-rtTA/alb-CRE /tet-YAP^{S127A}. “YAP binding” indicates the genes bound at the promoter (P) or at either the promoter or enhancers by YAP, based on ChIP-seq data of LaptTA/tet-YAP^{S127A} mice. “Gene signatures” show the MSigDB signatures that were used along with the indication of the genes included (the data used in this figure is reported in suppl. table 2 and 3). (**B**) Projection of adult hepatocytes signatures (AIZARANI C11 and C14) signatures and hepatoblast signatures in UMAPs reporting scRNA-seq of hepatocytes isolated from LaptTA/tet-YAP^{S127A} liver (as shown in figure 2 E).

Suppl figure 4

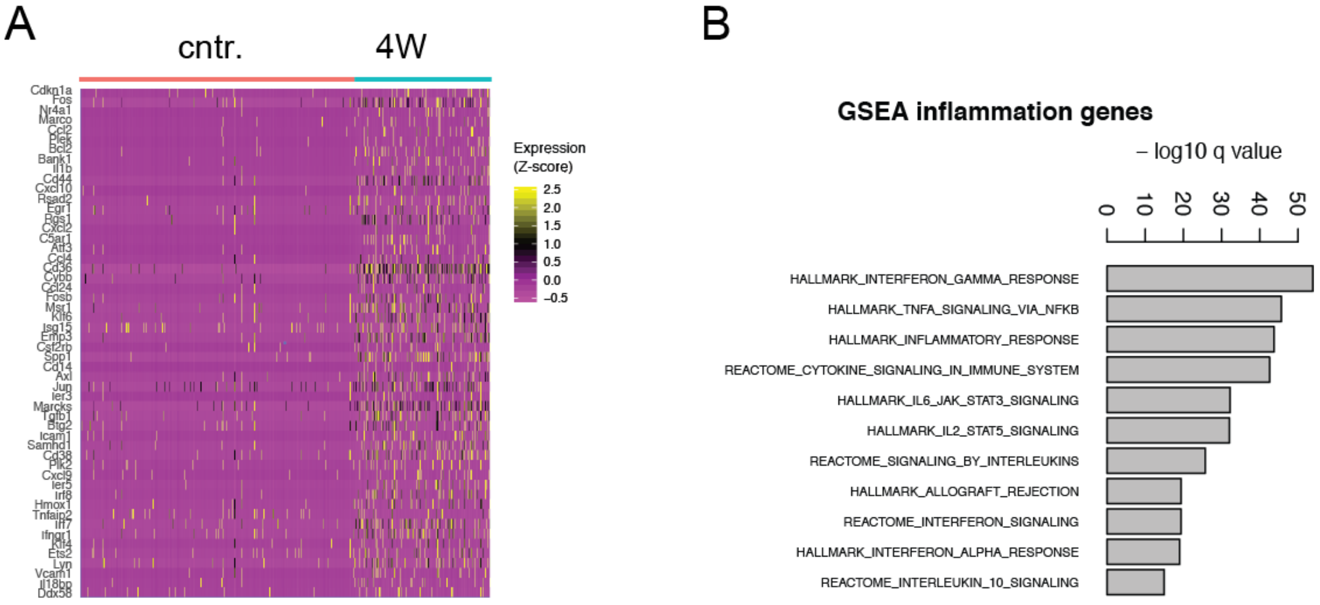

Supplementary Figure 4. Single-cell RNA-seq analysis of hepatocytes isolated from LaptTA/tet-YAP^{S127A} liver of either control animals (cntr.) or from transgenic mice following YAP^{S127A} induction for four weeks (4W). (**A**) Heatmap of the normalized expression of the inflammatory genes identified by scRNA-seq. (**B**) GSEA of the inflammatory genes identified by scRNA-seq as upregulated by YAP.

Suppl figure 5

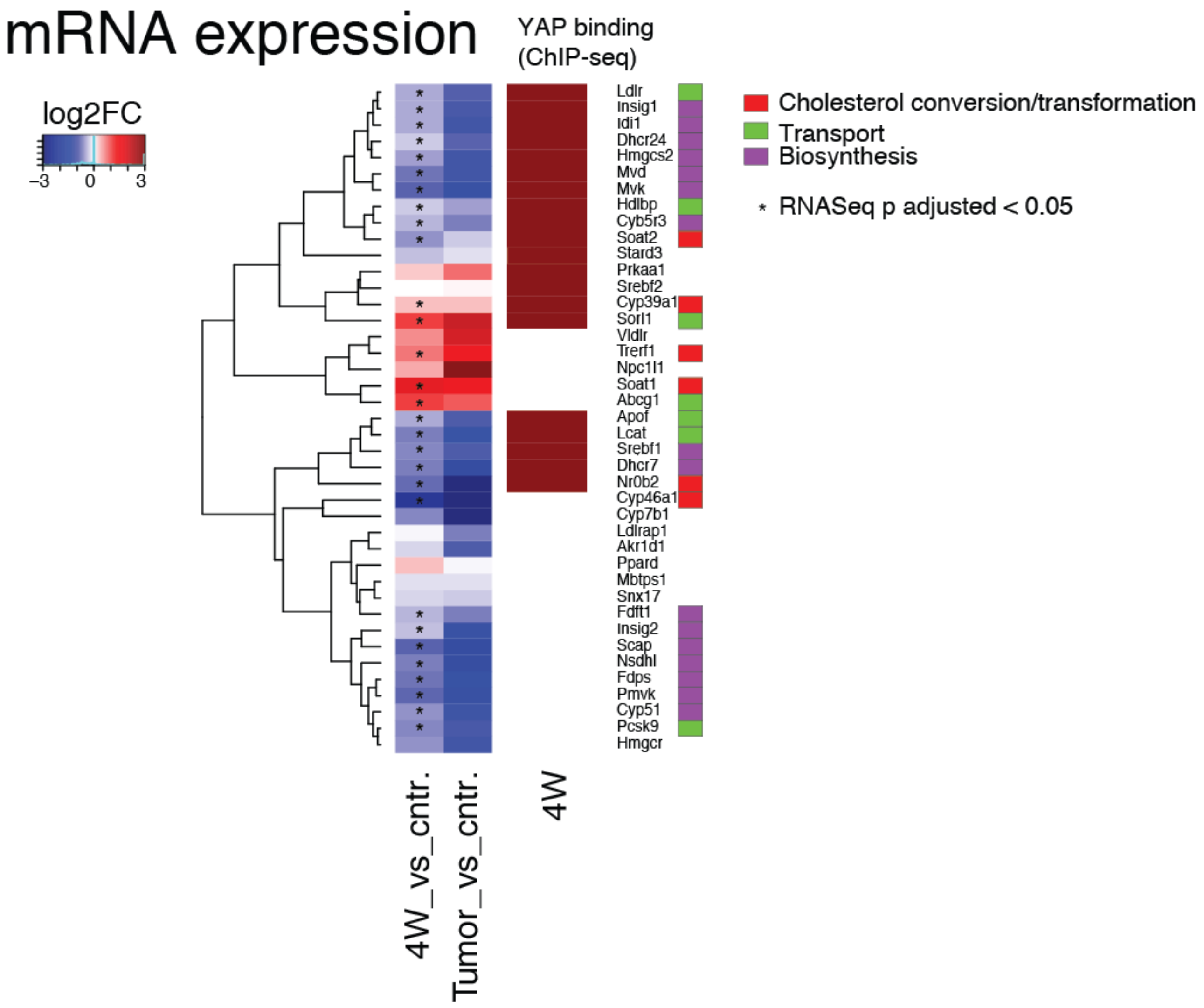

Supplementary Figure 5. Heatmap of the differential expression of genes involved in cholesterol metabolism.

Suppl figure 6

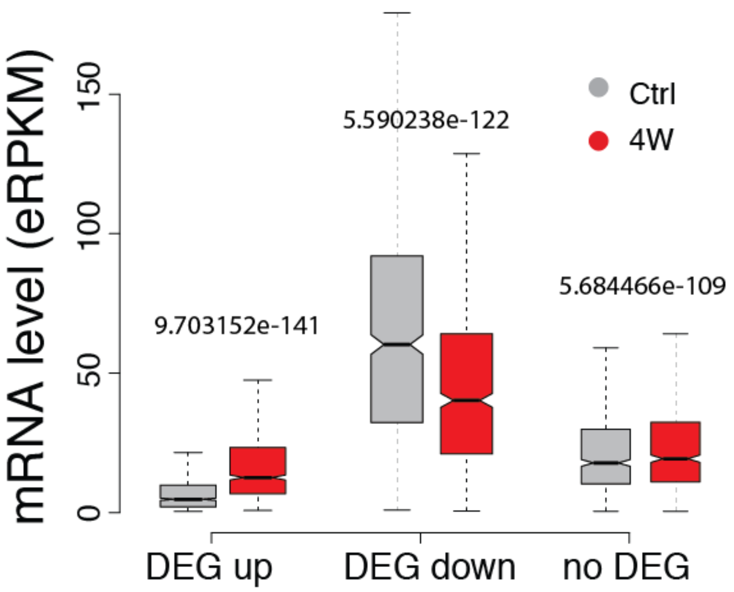

Supplementary Figure 6. Expression levels (eRPKM) of genes bound by YAP. Genes were subsetted in: up-regulated (DEG-up), down-regulated (DEG-down) or not changed (no-DEG) genes, based on their differential expression following YAP induction. Genes upregulated by YAP are lowly expressed in wild-type liver, while down-regulated genes are highly expressed. Numbers in box-plots indicated the p-value.

Suppl figure 7

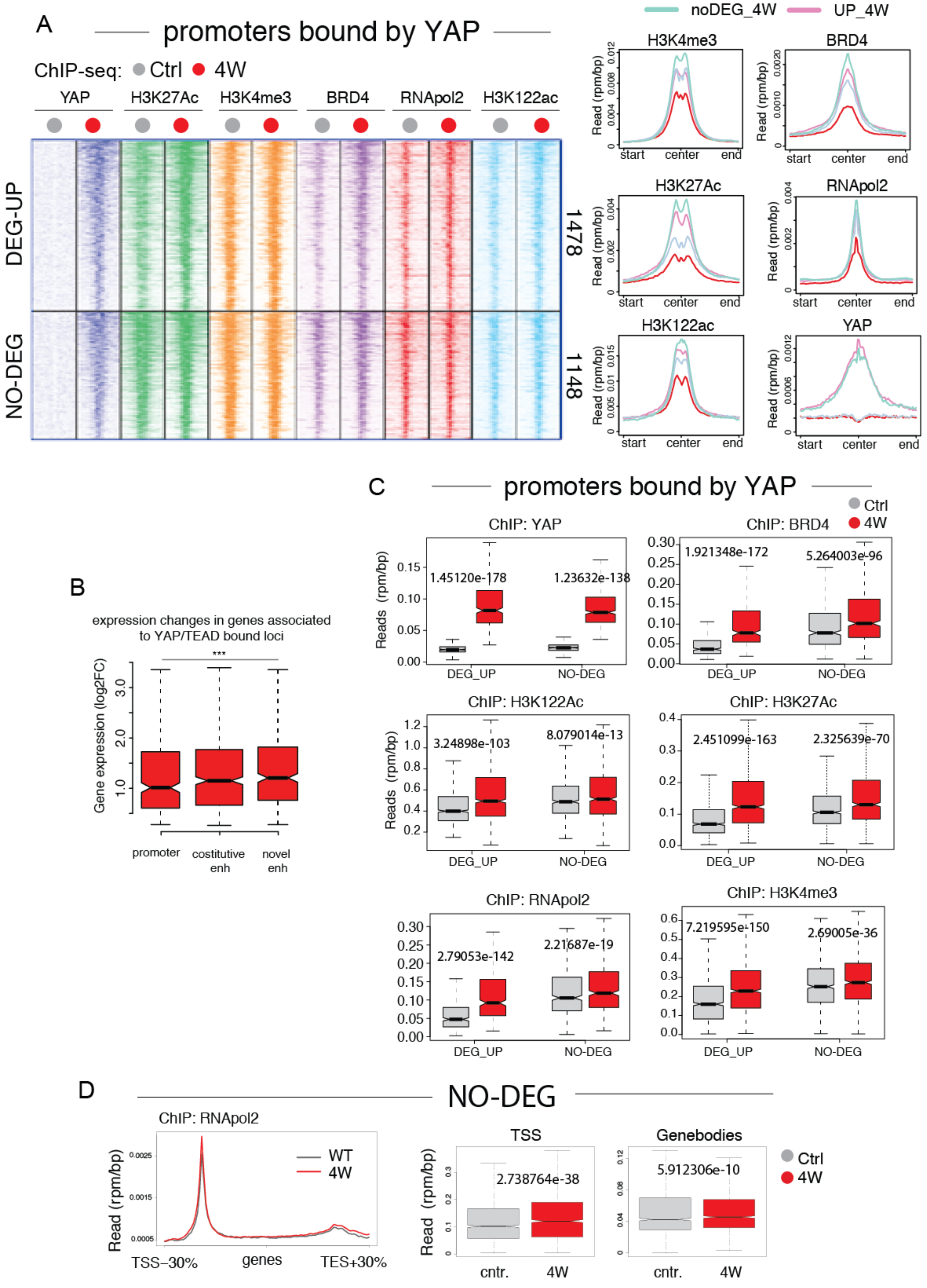

Figure legend is on the next page

Supplementary Figure 7. ChIP-seq analysis of hepatocytes isolated from LaptTA/tet-YAP^{S127A} liver of either control animals (cntr.) or following YAP^{S127A} induction for four weeks (4W). (**A**) Heatmap (left) and distribution-plots (right) of ChIP-seq signals found at promoters bound by YAP in LaptTA/tet-YAP^{S127A} liver. Promotes were subsetted in those associated to genes that were either up-regulated by YAP (DEG-UP) or did not change expression (NO-DEG). (**B**) Differential expression of DEG-UP genes bound by YAP at the promoter, constitutive enhancer (constit.) or novel enhancer (novel). Only the comparison promoter vs novel enhancers is statistically significant (mann withney test), Pval<0.001). (**C**) Box-plots of the ChIP-seq signals on promoters of genes bound by YAP that were either up-regulated (DEG-UP) or did not change expression (NO-DEG). (**D**) Left: metagene plot of RNApol2 ChIP-seq signals along genes that are bound by YAP at their promoter, but did not show a change in expression upon YAP induction. Right: RNApol2 ChIP-seq signals at transcription start sites (TSS) and along gene bodies (GB). Note that changes in chromatin marks and chromatin associated factors, were also noticeable (albeit with lower intensities) on the promoters of genes bound but not up-regulated by YAP: here YAP binding associated with a further increase in BRD4 and concomitant elevation of H3K122Ac. Yet these events did not associate with an increase in RNAPol2 recruitment and activity, conceivably because these genes were already transcribed at high levels (see Fig. S6). Numbers in box-plots indicated the p-value.

Suppl figure 8

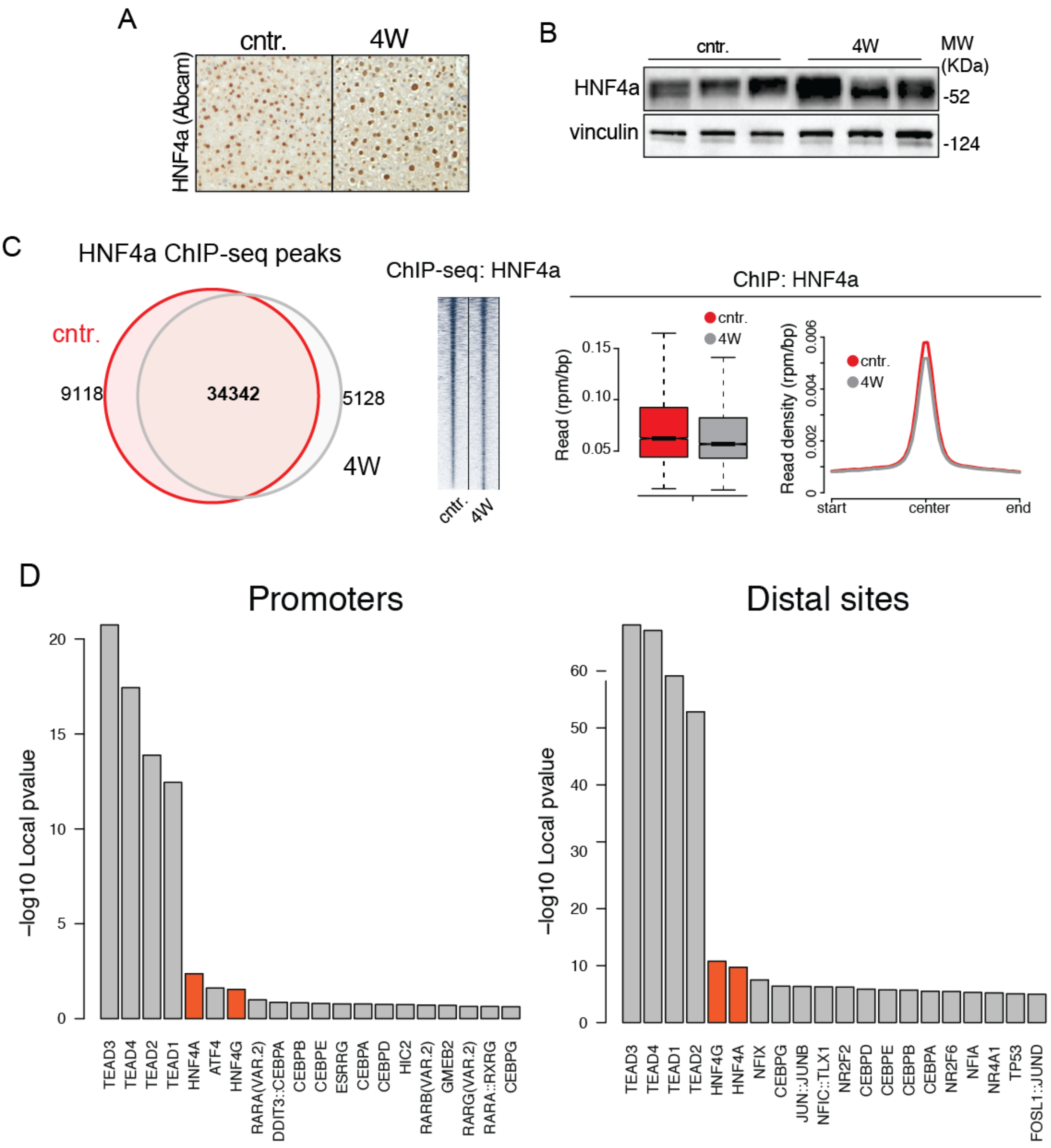

Supplementary Figure 8. Analysis of HNF4a in wild-type (cntr.) and in YAP expressing livers (4W= 4 weeks of induction) from LaptTA/tet-YAP^{S127A} mice. (**A**) IHC analysis of liver sections stained with an anti-HNF4a antibody. (**B**) Western-blotting analysis of liver protein extracts. Vinculin was used a loading control. (**C**) Heatmap, Venn diagram, box-plot and signal distribution of HNF4a ChIP-seq peaks. (**D**) TF binding motif analysis on promoters and distal sites of genes bound and down-regulated by YAP. Numbers in box-plots indicated the p-value.

Suppl figure 9

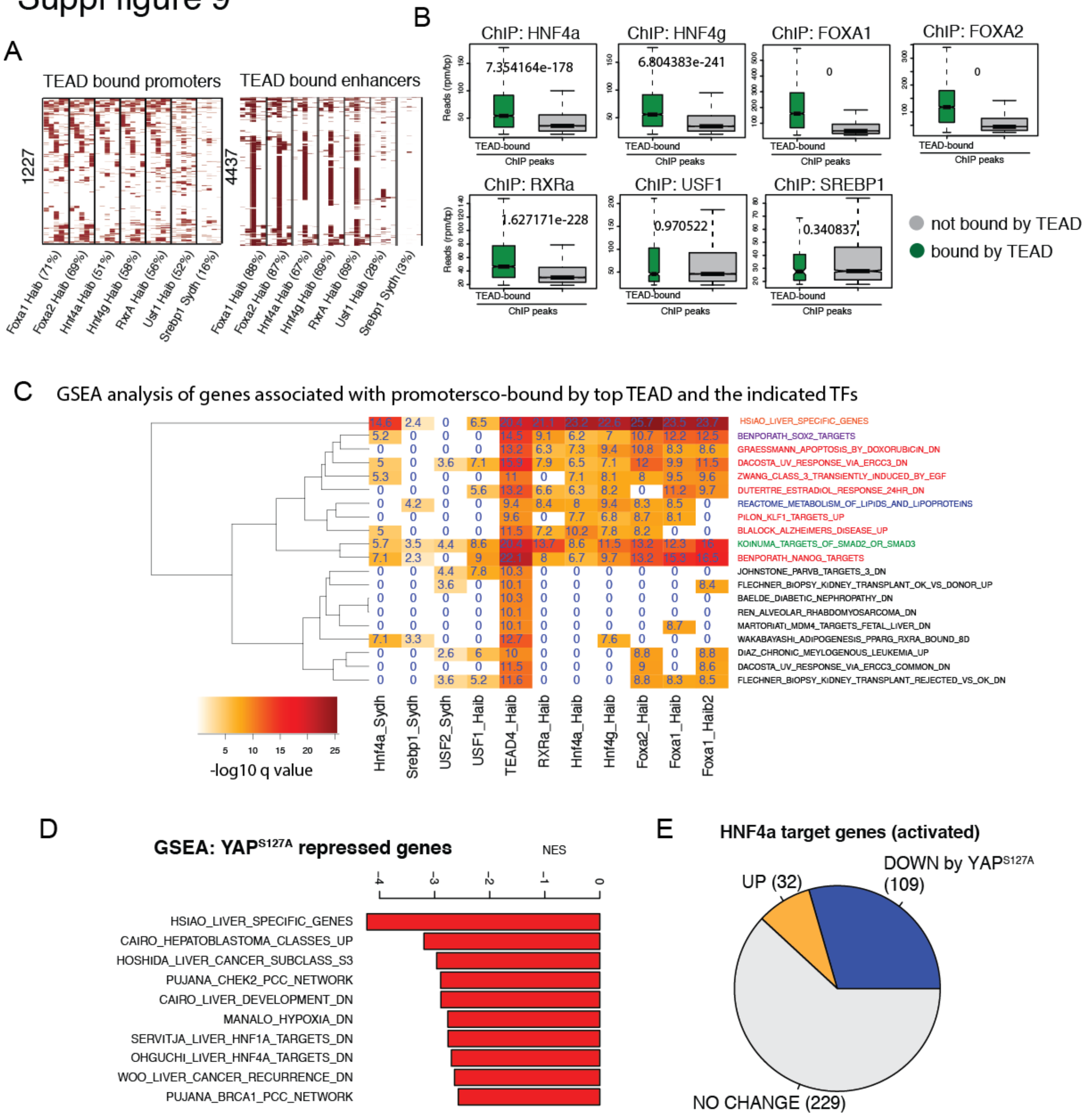

Supplementary Figure 9. Analysis of the Encode ChIP-seq data of HEPG2 cells. (**A**) Analogic heatmap of the ChIP-seq peaks found at promoters (left) and enhancers (right) bound by TEAD with high affinity (top 25%, ranked by TEAD ChIP-seq enrichment). Percentages are the fraction of high affinity TEAD peaks overlapping with the indicated transcription factors. (**B**) Box-plots of the ChIP-seq signals for the indicated transcription factors (TF). For each TF, peaks were sub-setted in TEAD bound (shown in green) and TEAD unbound (shown in gray). ChIP-seq enrichment of the majority of these TFs was higher on peaks co-localizing with TEAD, reinforcing a possible functional association of these TFs in the control of transcription. (**C**) Clustering based on the GSEA enrichment for the genes bound by the TF considered. This analysis shows that TFs co-localize with TEAD on genes linked to signalling (red), metabolic control in the liver (blue) and liver genes (orange). (**D**) GSEA analyses of genes downregulated by YAP^{S127A} in HEPG2 cells
(**E**) Venn diagram of overlap the HNF4a up-regulated genes and the YAP^{S127A} down-regulated genes identified in HEPG2 cells. Numbers in box-plots indicated the p-value.

Suppl figure 10

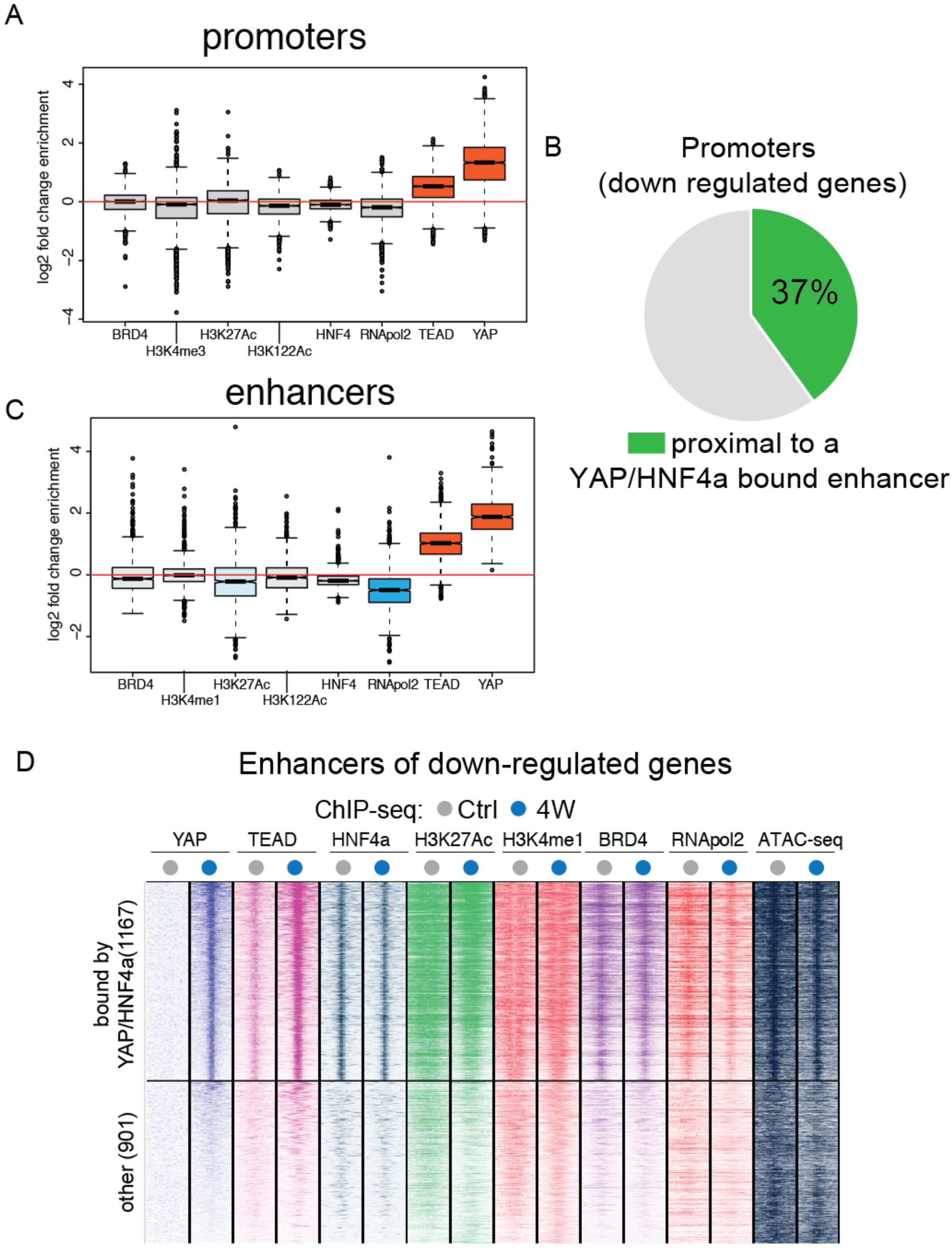

Supplementary Figure 10. ChIP-seq analysis of genes down-regulated upon the induction of YAP in LaptTA/tet-YAP^{S127A} livers for 4 week (4W). cntr.: wild-type mice.
(**A**) Box-plots of the differential enrichment of ChIP signals determined after YAP induction at promoters of down-regulated genes. This shows the lack of relevant alteration of HNF4a binding and chromatin marks due to YAP induction. (**B**) Pie chart of the fraction of down-regulated genes that have a promoter-proximal enhancer bound by both YAP and HNF4a. (**C**) Box-plots of the differential enrichment of ChIP signals determined after YAP induction at YAP/HNF4a bound enhancers proximal to down-regulated genes. This shows that major consequences of YAP binding are the increased recruitment of TEAD and the lowering of RNAPol2 and H3K27ac signals at YAP/HNF4a bound enhancers. (**D**) heatmap of the ChIP-seq signals for the enhancers proximal to YAP down-regulated genes. Enhancers proximal to down-regulated genes were split into YAP/HNF4a bound (1167 enhancers) and not bound (other, 901 enhancers).

Suppl figure 11

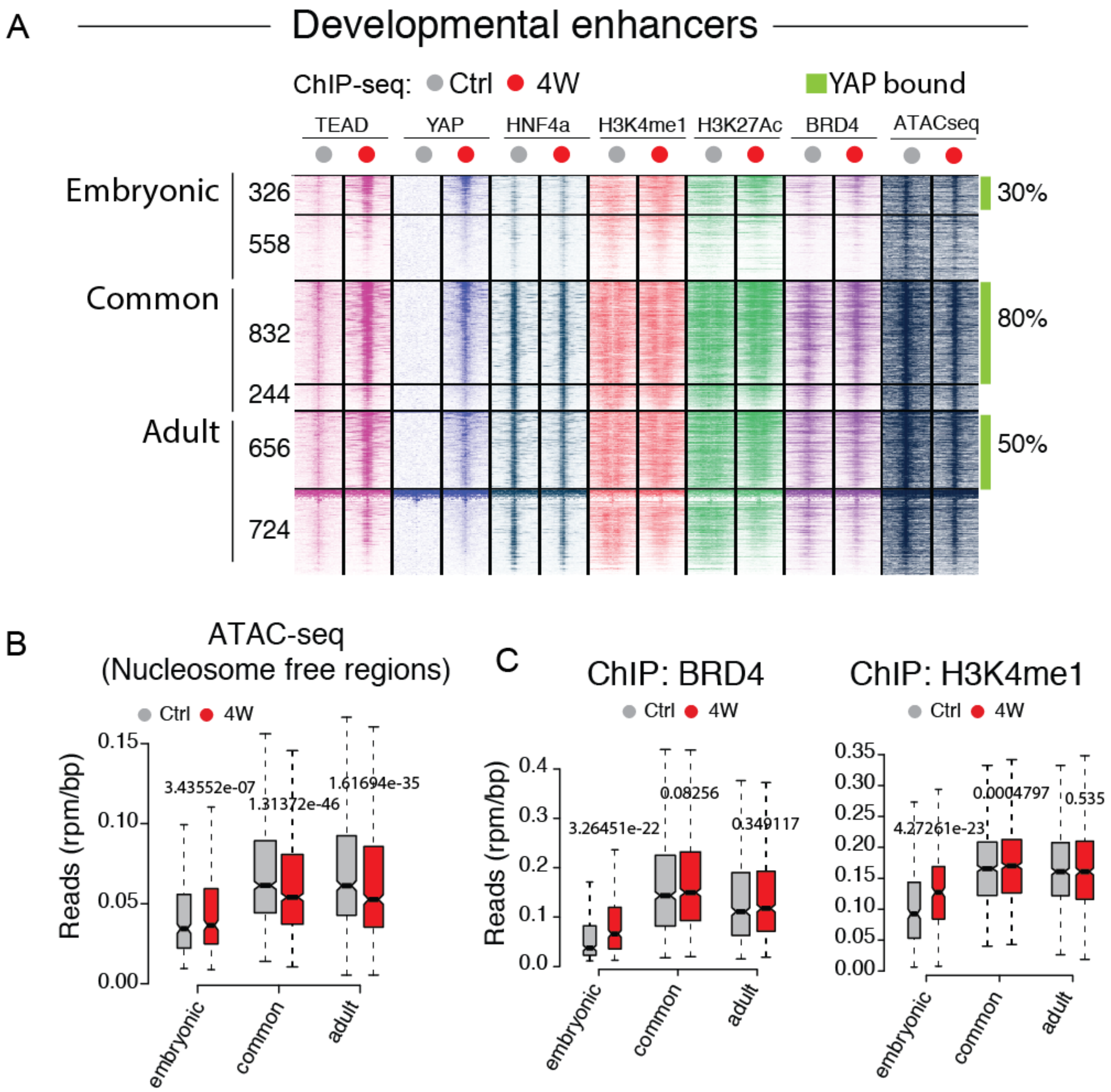

Supplementary Figure 11. ChIP-seq analysis of developmental enhancers identified in wild-type (cntr.) and YAP expressing livers (4W) from LaptTA/tet-YAP^{S127A} mice. Developmental enhancers were subsetted in embryonic, common and adult.
(**A**) Heatmap of the ChIP-seq signals. (**B**) Box-plot of nucleosome free signals determined by ATAC-seq. (**C**) Box-plots of ChIP-seq signals for BRD4 (left) and H3K4me1 (right). Numbers in box-plots indicated the p-value.

Suppl figure 12

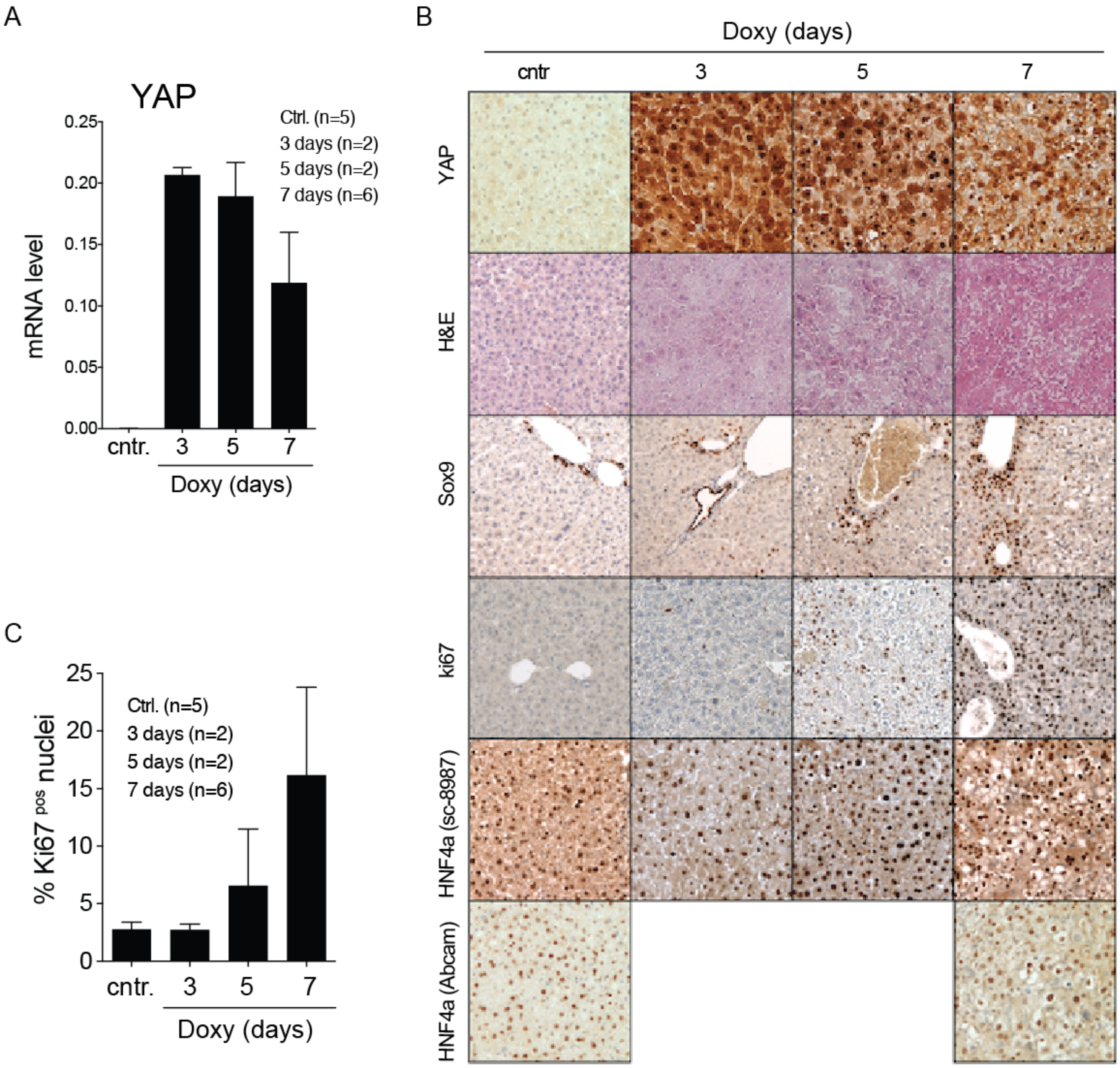

Supplementary Figure 12. Analysis of livers from R26-lsl-rtTA/alb-CRE/tet-YAP^{S127A} mice at different time points following YAP induction by doxycycline (doxy). Bar-plots show the average value, error bars indicate the standard deviation. (**A**) RT-qPCR analysis of livers processed at different time points following YAP induction. (**B**) IHC analysis of liver sections. Sections were stained with the indicated antibodies. H&E: hematoxylin and eosin staining. (**C**) Bar-plot of Ki67 positive nuclei in liver sections of control (cntr.) and tet-YAP mice.

Suppl figure 13

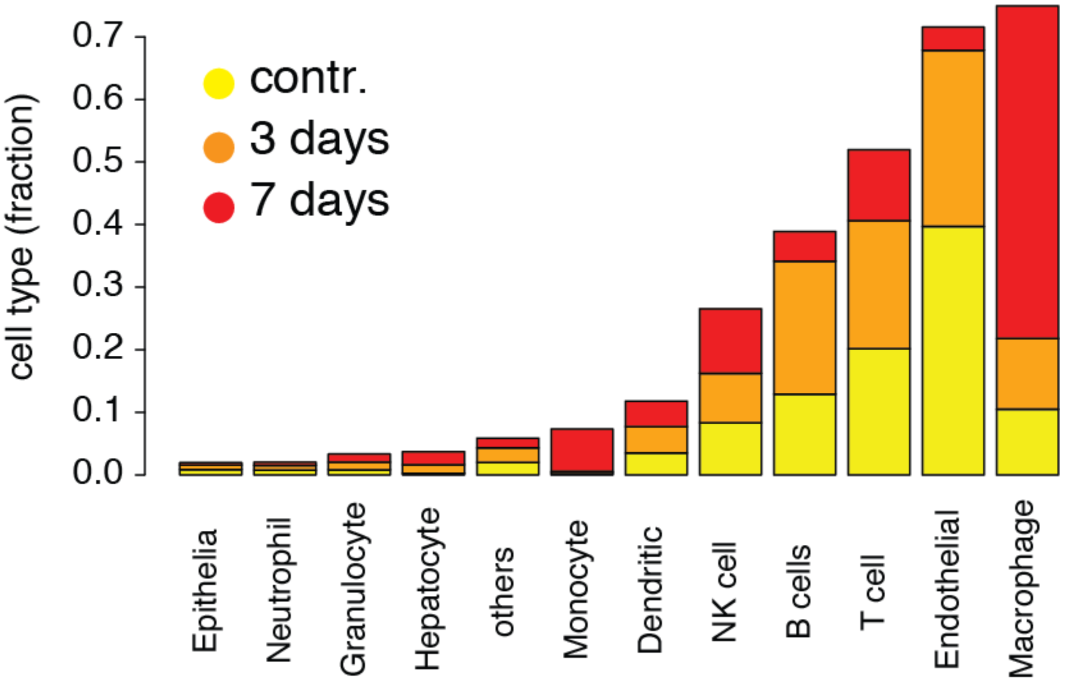

Supplementary Figure 13. Analysis of the cellular heterogeneity of the liver microenvironment of R26-lsl-rtTA/alb-CRE/tet-YAP^{S127A} liver cells by single-cell RNA-seq. The bar plot shows the relative fraction of the annotated cell type found at the different time points following YAP activation.

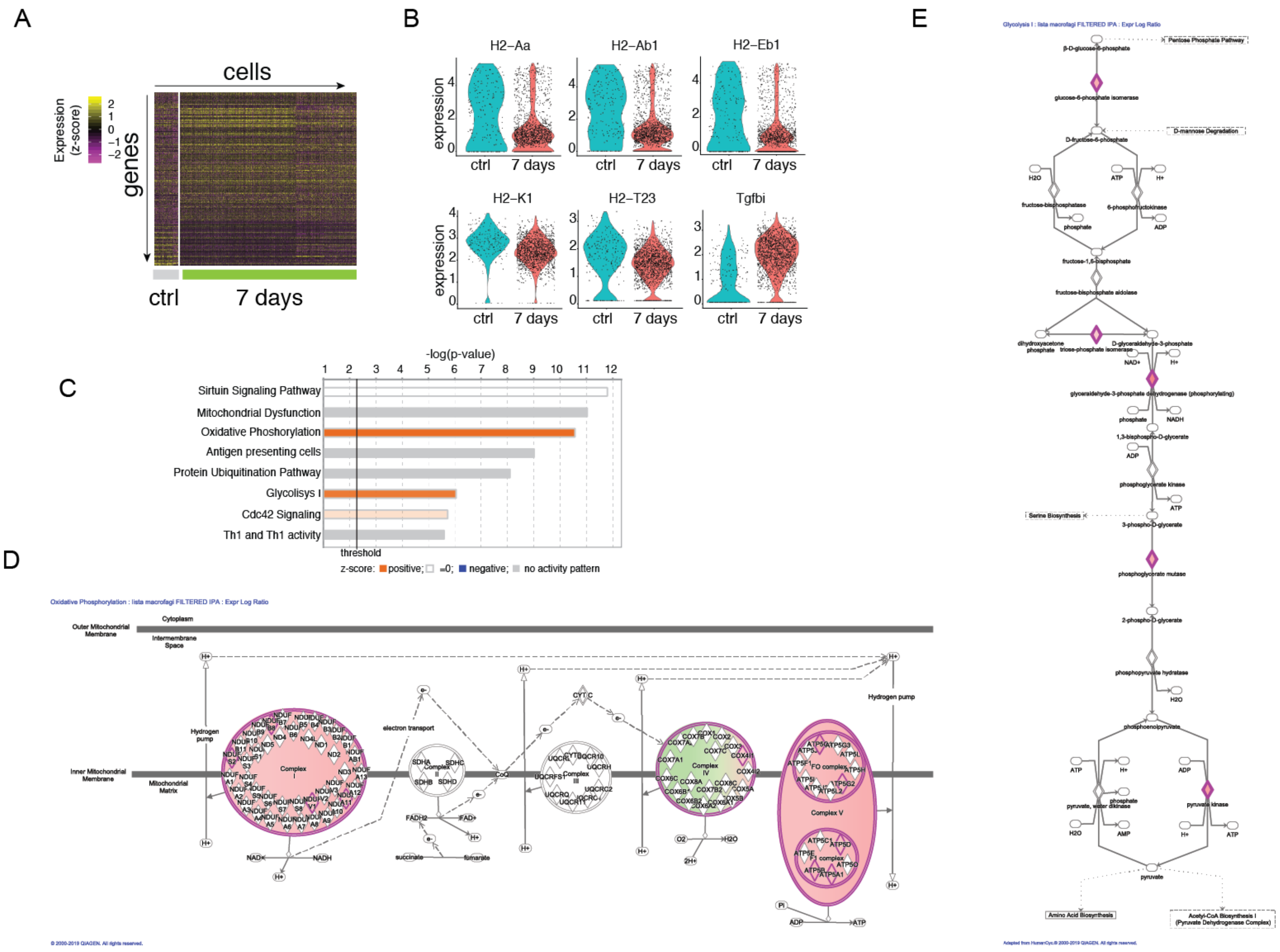

Supplementary Figure 14. scRNA-seq analysis of liver macrophages after 7 days of YAP induction in R26-lsl-rtTA/alb-CRE/tet-YAP^{S12A} mice.
(**A**) Heatmap of the differentially expressed genes identified in macrophages following YAP induction in hepatocytes.
(**B**) Violin plot of representative mRNAs differentially expressed in macrophages.
(**C**) IPA analysis of enriched pathway in differentially expressed genes shown in (A). Pathways with positive z-score (bioinformatic evidence of the activation of the pathway) are coloured in orange.
(**D**) Scheme of the mitochondrial oxidative phosphorylation. Components upregulated following YAP induction are outlined in purple.
(**E**) Scheme of the glycolytic pathway. Components upregulated following YAP induction are outlined in purple.

Suppl figure 15

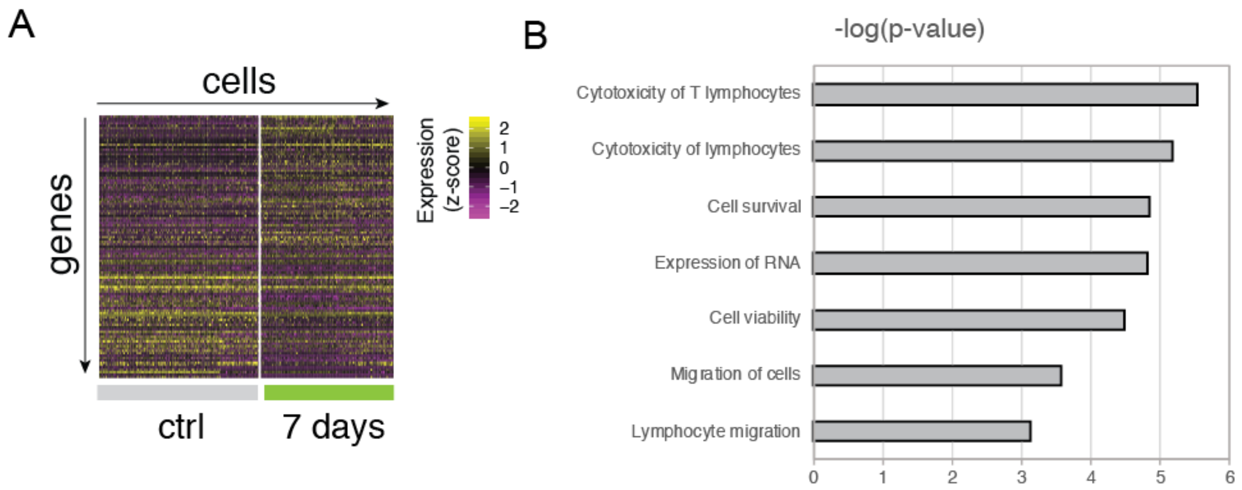

Supplementary Figure 15. scRNA-seq analysis of liver infiltrating T-lymphocytes after 7 days of YAP induction in R26-lsl-rtTA/alb-CRE/tet-YAP^{S127A} mice.

(**A**) Heatmap of the normalized expression (z-score) of the differentially expressed genes identified in T-lymphocytes, following YAP induction in hepatocytes.

(**B**) GSEA of genes down-regulated in T-lymphocytes following YAP activation (tet-YAP vs cntr.)

Suppl figure 16

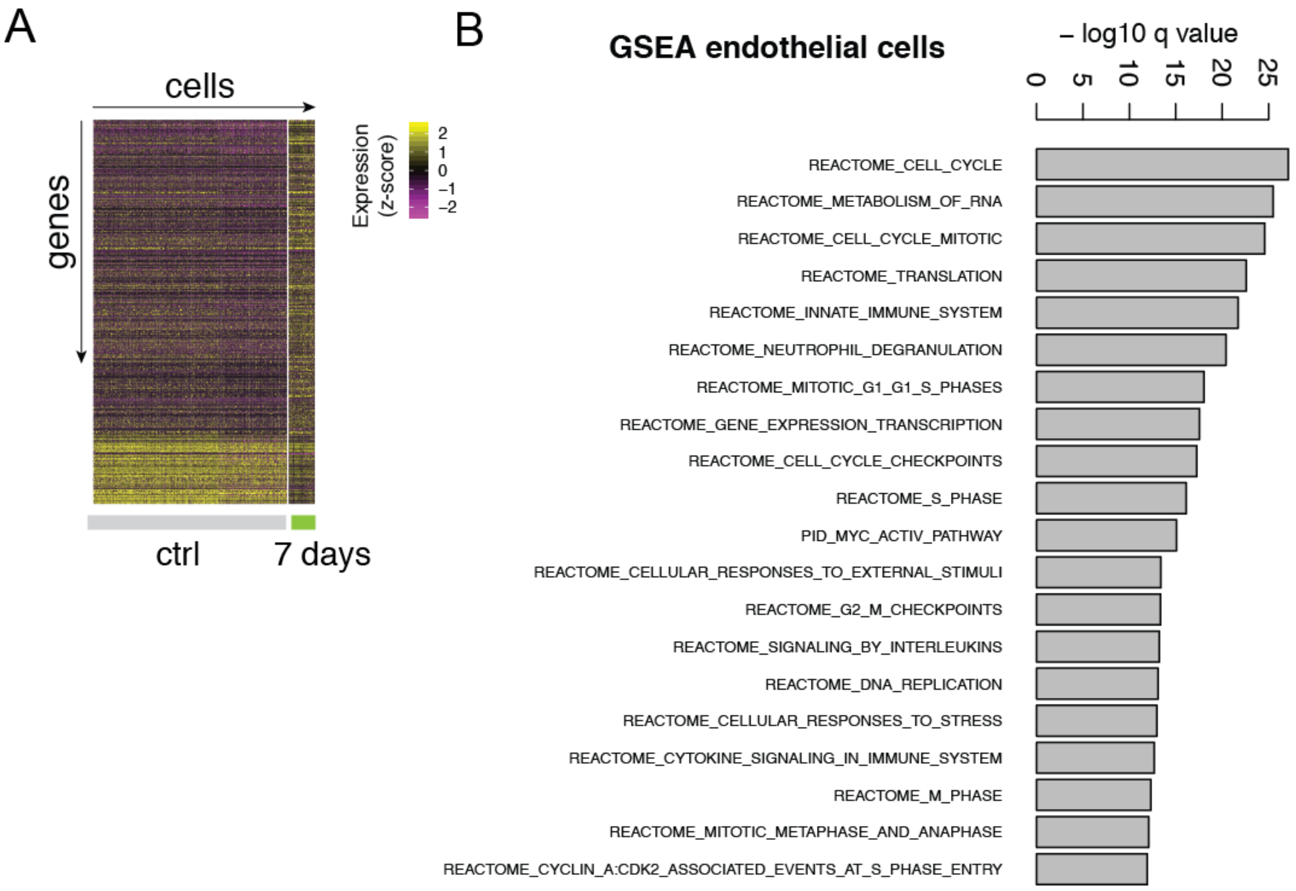

Supplementary Figure 16. scRNA-seq analysis of liver endothelial cells after 7 days of YAP induction in R26-lsl-rtTA/alb-CRE/tet-YAP^{S127A}.
(**A**) Heatmap of the normalized expression (z-score) of the differentially expressed genes identified in endothelial cells, following YAP induction in hepatocytes.
(**B**) GSEA of differentially expressed genes in endothelial cells (tet-YAP vs cntr.).

SUPPLEMENTARY MATERIALS AND METHODS

Reagents, plasmid, and transfection. Doxycycline Hyclate was purchased from Sigma, (D9891-1G). pSLIK-YAP^{S127A} vector was generated as in Croci et al.(1).

For vectors used in Luciferase assay, we proceeded to amplify Apob promoter, Apob enhancer#1, and enhancer#2 using MCF10a genomic DNA as template and primers in Supplementary Table S2. The destination vector chosen was pGL3 basic (Promega, E1751). The Renilla TK vector (Promega, E2241) was used to normalize the Luciferase signal. Plasmids were transfected with Lipofectamine 3000 (Life Technologies L3000015) in an antibiotics-free medium according to manufacturer instructions.

Human HNF4a siRNAs are SilencerTM Pre-Designed siRNAs from ThermoScientific (siHNF4a #1 -> clone ID 144546; siHNF4a#2 -> clone ID 290203) as SilencerTM Negative control no.1 siRNA (AM4611). siRNA transfections were done with Lipofectamine RNAi-MAX (Life Technologies, #13778150) in antibiotics-free medium according to manufacturer instructions.

Cell culture. HEPG2 human hepatocellular cell line (ATCC) was cultured in RPMI supplemented with 10% FBS, 2mM L-glutamine, and 1% penicillin/streptomycin.

The cells were infected with pSLIK-YAP^{S127A} lentivirus and selected with 100 µg/mL Hygromycin, for one week. For subsequent experiments, cells were grown in RPMI medium supplemented with 10% tetracyclin-free serum (Hyclone SH30070). YAP^{S127A} expression was induced by 2 µg/ml doxycycline. The HEPG2 cell line tested negative for Mycoplasma.

Luciferase reporter assay. A renilla-luciferase reporter assay was carried out to examine the effect of YAP^{S127A} overexpression on ApoB promoter and enhancers activity. A total of 10^4 HEPG2 pSLIKYAP^{S127A} cells were seeded on a 12-well plate in antibiotic-free media for 24 hours. Cells were transfected with 5 ng TK Renilla vector (used as an internal control) and 100 ng of either pGL3-ApoB promoter, or pGL3-ApoB enhancer#1 and enhancer#2 vectors. 24h after transfection cells were treated with 2 µg/ml doxycycline for an additional 24 hours to induce YAP^{S127A} expression. Then cells were harvested and lysed. Luciferase activity was tested using the Dual-Luciferase Reporter Assay System (Promega E1960) as instructed by the manufacturer and luciferase detection was measured on GloMax® Explorer Multimode Microplate Reader (Promega).

Western Blot. Cells or liver tissues were lysed with NP40 buffer (150 mM NaCl, 50 mM Tris pH 8.0, 1 mM EDTA, 1% NP40) supplemented with MINI-complete Protease Inhibitor Cocktail Tablets (Roche, #11836153001) and phosphatase inhibition PhosSTOP (Roche, #4906845001) and sonicated (Branson). Cleared lysates were quantified by Bradford assay, run on a TGX Precast Protein Gels (BioRad) and immunoblotted with the indicated primary

antibodies. Chemiluminescent detection after incubation of the membranes with the appropriate secondary antibody was done using the ChemiDoc System (Bio-Rad).

Hepatocytes isolation. Hepatocytes isolation was obtained by modifying a previously established, two-step liver perfusion protocol(2). Mice were euthanized, the liver was surgically exposed and a cannula was inserted into the cava vein. The catheter was connected to the pump and immediately the portal vein was cut. Mouse livers were then perfused by a pre-warmed (37°C) perfusion buffer (50 mM EDTA in 1X EBSS without Ca, Mg, Phenol Red [GIBCO, 14155-048]) at an initial flow rate of 8 ml/min for 3 minutes. This was then followed by perfusion with a pre-warmed (37°C) Collagenase Type II (50U/ml; Worthington, LS004174) digestion buffer solution (10 mM HEPES in 1x EBSS with Ca, Mg, Phenol Red [GIBCO, 24010-043]) for 8-10 minutes at a flow rate of 5 ml/min. After isolating the liver and surgically removing the gallbladder, the liver was gently mechanically dissociated in Collagenase Type II (35 U/mL) digestion buffer solution (10 mM HEPES in 1x EBSS with Ca, Mg, Phenol Red) until reaching single-cell suspension. Dissociated cells were filtered through a 100 μm cell strainer and washed twice. After the last wash, cells were resuspended in cold DMEM, 10% FBS and counted with Trypan Blue.

Single cells isolation from the liver. Isolation of single cells (non-hepatocytes) from the liver was based on Bisso et al.(3). Briefly, liver was removed and minced with a scalpel into small pieces. Tissue fragments were digested with 0.5 U/ml dispase (Stemcell technologies #07913) and 0.1 U/ml collagenase (Sigma-Aldrich, #C2674) for 30 minutes at 37°C, in DMEM medium with gentle shaking. Cells were passed through a 70 μm nylon mesh filter (Falcon #352350), centrifuged at 80g for 10 minutes, washed once with PBS, counted and resuspended at the correct concentration for single cell GEMs preparation.

Immunohistochemical analysis. Fresh livers were fixed with 4% formaldehyde in PBS, (overnight at 4°C), paraffin-embedded and sectioned at 6-8 μM of thickness. For histology, sections were counter-stained with Haematoxylin and eosin. Immunohistochemistry was performed as previously described(4).

RNA isolation. Total RNA from HEPG2 cell line or liver samples was extracted with Quick RNA miniprep kit from Zymo Research (R1055) following manufacturer instruction. The RNA concentration was assessed by Nanodrop and then it was stored at -80°C.

RNA retrotranscription and Quantitative Real-Time PCR (RT-qPCR). RNA retrotranscription was performed using the ImProm-II Reverse Transcription System (Promega, A3800), following manufacturer instructions. qPCR was performed in a total volume of 20 μl with SsoAdvanced Universal SYBR Green Supermix (BioRad, #172-5272) and run on CFX Connect Real-Time PCR Detection System (BioRad). qPCR was performed in triplicate and all data were normalized to RPPO. PCR oligo sequences are listed in

Supplementary Table S3. Error bars in graphs refer to a standard deviation on technical repeats.

RNA-sequencing. For RNA-Seq experiments, liver samples were firstly homogenized with GentleMacs Tissue Homogenizer (Miltenyi Biotec) and total RNA was purified as described above. Library preparation was performed with the TruSeq RNA Sample Prep Kits v2 (Illumina) following manufacturer instructions. RNA-Seq libraries were then run on the Agilent 2100 Bioanalyzer (Agilent High Sensitivity DNA chip) for quantification and quality control and then sequenced on Illumina NovaSeq 6000.

Single-cell RNA sequencing. Purified hepatocytes(2) or single cell extracts from liver were processed with the GemCode Single Cell Platform loaded into Chromium microfluidic chips. RNA from the barcoded cells was subsequently reverse-transcribed and sequencing libraries were prepared with Chromium Single Cell v2 and v3 reagent kit (10X Genomics). Sequencing was performed with Illumina NovaSeq 6000 according to the manufacturer's instructions (Illumina).

Bioinformatic analyses

Row NGS-data processing. ChIP-Seq single-end reads, ATAC-Seq paired-end reads and bulk RNA-Seq paired-end reads were processed using HTS-flow framework(5).

Read filtering. Quality of the raw reads was assessed using FastQC program (http://www.bioinformatics.babraham.ac.uk/projects/fastqc/), while filtering of low-quality reads was done using fastq_masker (options –Q 33 –q 20 –r –N –v -i) (http://hannonlab.cshl.edu/fastx_toolkit/).

ChIP-Seq analyses. Filtered reads were aligned to the mouse genome (mm9 genome assembly) using BWA-MEM v. 0.6.2 program(6). Bam files of the replicates were merged together, and duplicates were removed. Peaks were called using MACS v. 1.4(7); for YAP and TEAD at 4W in LapTTA system, using a p-value threshold of 10-5; peaks of other transcription factor were called with MACS v. 2 and a p-value threshold of 10-5, while histone modifications (H3K4me1, H3K27Ac) peaks were called with MACS v. 2 and a p-value threshold of 10-8. Input for peak calling and peaks in WT liver were taken from GEO published data with accession number GSE83863.

Coordinates of Hnf4a and Foxa2 peaks in embryonic and adult liver to obtain embryonic, common and adult enhancers were kindly provided by Pamela Hoodless(8).

HepG2 peaks were downloaded from ENCODE(9) (name of the files:
wgEncodeAwgTfbsHaibHepg2Foxa1sc6553V0416101UniPk.narrowPeak
wgEncodeAwgTfbsHaibHepg2Foxa2sc6554V0416101UniPk.narrowPeak
wgEncodeAwgTfbsHaibHepg2Hnf4asc8987V0416101UniPk.narrowPeak
wgEncodeAwgTfbsHaibHepg2Hnf4gsc6558V0416101UniPk.narrowPeak

wgEncodeAwgTfbsHaibHepg2RxraPcr1xUniPk.narrowPeak
wgEncodeAwgTfbsHaibHepg2Tead4sc101184V0422111UniPk.narrowPeak
wgEncodeAwgTfbsHaibHepg2Usf1Pcr1xUniPk.narrowPeak
wgEncodeAwgTfbsSydhHepg2Srebp1InslnUniPk.narrowPeak)

Reads count of a ChIP for a specific genomic region was calculated as the sums of the pileups of the reads for each base pair inside that genomic region; this number was normalized by the library size of the ChIP, obtaining the reads per million (rpm). H3K122Ac signals were normalized using all the reads aligned to all annotated promoters of mm9 instead of the total library size. Read density (rpm/bp) for specific genomic regions was obtained by dividing the normalized reads (rpm) by the number of the base pair of the region. Computation of reads enrichment, overlaps, visualization of the results was carried out using custom R scripts, with R version 3.5.1 and using the functions of compEpiTools package(10).

Bulk RNA-Seq analyses. Filtered reads were aligned to mouse genome (mm9 genome assembly) using tophat v. 2.0.8(11) with the options --r 170 –p 8 --no-novel-juncs --no-novel-indels --library-type fr-unstranded. Differential gene expression was carried out with DESeq2 R Bioconductor package(12). Genes were called as “up-regulated” (DEG up) when p adjusted was < 0.01 and log2 fold change in gene expression was > 0.5; “down-regulated” (DEG down) if p adjusted was < 0.01 and log2 fold change was < -0.5; “not deregulated” (no DEG) if |log2 fold change| was <0.1. For fig. 4 (normalized enrichment score from gene-set enrichment analysis), the log2 fold change thresholds for DEG up and DEG down was set to +1 and -1, respectively.

ATAC-Seq analyses. Filtered reads were aligned to mouse genome (mm9 genome assembly) using BWA-MEM v. 0.6.2 program(6). Reads from the replicates were merged together, and duplicates were removed (obtaining >100 million aligned reads for both samples). Fragment size of alignments were then assessed with Rsamtools R package (http://bioconductor.org/packages/Rsamtools). Next, bam files were splitted according to the fragment size distribution using alignmentSieve function from deepTools package(13): reads of fragments <90 nt were extracted in a dedicated bam file and considered as belonging to “nucleosome-free regions”.

Single-cell RNA-Seq analyses. Reads were processed using cellranger v. 2.1.1 for LSL cells (entire liver tissue, 3’ v2 libraries) and cellranger v. 3.0.0 for perfused liver (3’ v3 libraries) (https://support.10xgenomics.com/single-cell-gene-expression/software/overview/welcome) with default parameters (Chromium 10X pipeline); reads were aligned to mm10 genome assembly and feature/barcode matrixes were obtained. All subsequent analyses were carried out using Seurat R package(14, 15) : version 2 was used for LSL cells (entire liver), while version 3 for perfused liver samples.

Replicates of the samples obtained from entire tissue (LSL system) were merged together and t-distributed stochastic neighbor embedding (t-SNE) was produced for visualization. Cells obtained from perfused liver were down-sampled to 3000 cells for each condition, and cells having a number of unique molecular identifiers (UMI) > 7500 were excluded from the analyses and uniform manifold approximation and projection (UMAP) was produced for visualization. For the identification of cell-type scMCA R package was used(16); expression of gene signatures was calculated with the average Z-scores of all the genes belonging to that signature.

Definition of promoter, intragenic and intergenic regions. Peaks or any genomic region were considered as belonging to promoters if they overlapped with at least 1 base pair with the interval [-2000;+1000] from annotated TSS (transcription start site). They were considered belonging to gene bodies ("intragenic") if they were not at promoters but overlapped with at least 1 base pair with any annotated transcripts. The rest of the regions (neither at promoters nor intragenic) were considered "intergenic" regions. Annotation was carried out using TxDb.Musculus.UCSC.mm9.knownGene R package from Bioconductor(17).

Motif finding and gene set enrichment analyses (GSEA). Motif finding analyses on genes downregulated was performed with PscanChIP tool(18) (http://159.149.160.88/pscan_chip_dev/). Briefly, Yap peak summits were associated to promoters of DEG down if they overlapped with [-2000;+1000] window from TSS of DEG down and they were associated to enhancers of DEG down if they overlapped with [-20000;-2000]U[+1000;+20000] window from TSS of DEG down. The following parameters were set for the analysis: I) genome assembly: mm9 II) Background: “mixed” for enhancers, “promoters” for promoters III) Database: Jaspar 2018(19). Gene set enrichment analyses (fig. 4) were carried out using, as input, DEG up and DEG down called with the following thresholds: |log2 fold change|>1 and p adjusted <0.01, using GSEA 3.0 pre-ranked java application(20) (and “Hallmarks” and “C2 curated” genesets from MSigDB(21). For the statistics, 10000 random permutations were set as parameters in the application and only genesets with p adjusted < 0.05 were considered as significant. To identify biological functions affected by YAP overexpression on macrophages cell population, we used Ingenuity Pathway Analysis (IPA, Ingenuity®Systems). The log2 fold change data for deregulated genes were tabulated and uploaded into the IPA web application (www.ingenuity.com) as indicated in Supplementary Tables 11, 12 and 13. We used a z-score algorithm to identify the biological functions increased or decreased according to the gene expression changes in our dataset.

YAP target gene signatures. Signature of YAP regulated genes identified by RNA-seq shown in figure 2 were derived by defining DEGs as genes with log2FC above (DEG-Up) or

below (DEG-Down) of 0.5 and a P adj. < 0.01. DEGs were then annotated using GSEA signatures as follows: "Inflammation-Up" are DEG-Up genes found in HALLMARK_INFLAMMATORY_RESPONSE,

HALLMARK_TNFA_SIGNALING_VIA_NFKB

HALLMARK_IL6_JAK_STAT3_SIGNALING

HALLMARK_IL2_STAT5_SIGNALING

HALLMARK_INTERFERON_GAMMA_RESPONSE

"Cell Cycle UP" are DEG-Up genes found in:

KONG_E2F3_TARGETS,

FISCHER_G2_M_CELL_CYCLE

KEGG_CELL_CYCLE

HALLMARK_G2M_CHECKPOINT

HALLMARK_E2F_TARGETS

HALLMARK_MITOTIC_SPINDLE

HALLMARK_KRAS_SIGNALING_UP

"HNF4a_targets_down" were all genes bound by HNF4 and YAP (4W condition) at their promoters ([-2000;+1000] from TSS) and that were down-regulated by YAP (padj<0.01 and log2FC < -0.05). "HNF4a_targets_up" were defined as, but with log2FC > 0.05.

Supplementary References

1. Croci O, De Fazio S, Biagioni F, Donato E, Caganova M, Curti L, Doni M, et al. Transcriptional integration of mitogenic and mechanical signals by Myc and YAP. Genes Dev 2017;31:2017-2022.
2. Li WC, Ralphs KL, Tosh D. Isolation and culture of adult mouse hepatocytes. Methods Mol Biol 2010;633:185-196.
3. Bisso A, Filipuzzi M, Gamarra Figueroa GP, Brumana G, Biagioni F, Doni M, Ceccotti G, et al. Cooperation Between MYC and beta-Catenin in Liver Tumorigenesis Requires Yap/Taz. Hepatology 2020;72:1430-1443.
4. Campaner S, Doni M, Hydbring P, Verrecchia A, Bianchi L, Sardella D, Schleker T, et al. Cdk2 suppresses cellular senescence induced by the c-myc oncogene. Nat Cell Biol 2010;12:54-59; sup pp 51-14.
5. Bianchi V, Ceol A, Ogier AG, de Pretis S, Galeota E, Kishore K, Bora P, et al. Integrated Systems for NGS Data Management and Analysis: Open Issues and Available Solutions. Front Genet 2016;7:75.
6. Li H, Durbin R. Fast and accurate short read alignment with Burrows-Wheeler transform. Bioinformatics 2009;25:1754-1760.
7. Zhang Y, Liu T, Meyer CA, Eeckhoute J, Johnson DS, Bernstein BE, Nusbaum C, et al. Model-based analysis of ChIP-Seq (MACS). Genome Biol 2008;9:R137.
8. Alder O, Cullum R, Lee S, Kan AC, Wei W, Yi Y, Garside VC, et al. Hippo signaling influences HNF4A and FOXA2 enhancer switching during hepatocyte differentiation. Cell Rep 2014;9:261-271.
9. Consortium EP. An integrated encyclopedia of DNA elements in the human genome. Nature 2012;489:57-74.

10. Kishore K, de Pretis S, Lister R, Morelli MJ, Bianchi V, Amati B, Ecker JR, et al. methylPipe and compEpiTools: a suite of R packages for the integrative analysis of epigenomics data. BMC Bioinformatics 2015;16:313.
11. Trapnell C, Pachter L, Salzberg SL. TopHat: discovering splice junctions with RNA-Seq. Bioinformatics 2009;25:1105-1111.
12. Love MI, Huber W, Anders S. Moderated estimation of fold change and dispersion for RNA-seq data with DESeq2. Genome Biol 2014;15:550.
13. Ramirez F, Ryan DP, Gruning B, Bhardwaj V, Kilpert F, Richter AS, Heyne S, et al. deepTools2: a next generation web server for deep-sequencing data analysis. Nucleic Acids Res 2016;44:W160-165.
14. Butler A, Hoffman P, Smibert P, Papalexi E, Satija R. Integrating single-cell transcriptomic data across different conditions, technologies, and species. Nat Biotechnol 2018;36:411-420.
15. Stuart T, Butler A, Hoffman P, Hafemeister C, Papalexi E, Mauck WM, 3rd, Hao Y, et al. Comprehensive Integration of Single-Cell Data. Cell 2019;177:1888-1902 e1821.
16. Sun H, Zhou Y, Fei L, Chen H, Guo G. scMCA: A Tool to Define Mouse Cell Types Based on Single-Cell Digital Expression. Methods Mol Biol 2019;1935:91-96.
17. Huber W, Carey VJ, Gentleman R, Anders S, Carlson M, Carvalho BS, Bravo HC, et al. Orchestrating high-throughput genomic analysis with Bioconductor. Nat Methods 2015;12:115-121.
18. Zambelli F, Pesole G, Pavesi G. PscanChIP: Finding over-represented transcription factor-binding site motifs and their correlations in sequences from ChIP-Seq experiments. Nucleic Acids Res 2013;41:W535-543.
19. Khan A, Fornes O, Stigliani A, Gheorghe M, Castro-Mondragon JA, van der Lee R, Bessy A, et al. JASPAR 2018: update of the open-access database of transcription factor binding profiles and its web framework. Nucleic Acids Res 2018;46:D1284.
20. Subramanian A, Tamayo P, Mootha VK, Mukherjee S, Ebert BL, Gillette MA, Paulovich A, et al. Gene set enrichment analysis: a knowledge-based approach for interpreting genome-wide expression profiles. Proc Natl Acad Sci U S A 2005;102:15545-15550.
21. Liberzon A, Subramanian A, Pinchback R, Thorvaldsdottir H, Tamayo P, Mesirov JP. Molecular signatures database (MSigDB) 3.0. Bioinformatics 2011;27:1739-1740.
